# Integrated Metabolic, Oxidative, Inflammatory, and Mammary Health Signatures Characterize Low Body Condition Score in Dairy Cows

**DOI:** 10.3390/ijms27167416

**Published:** 2026-08-19

**Authors:** Kamila Puppel, Jan Slósarz, Grzegorz Grodkowski, Paweł Solarczyk, Piotr Kostusiak, Małgorzata Kunowska-Slósarz, Karol Tucki, Krzysztof Młynek, Marek Balcerak

**Affiliations:** 1 Institute of Animal Science, Warsaw University of Life Sciences, Ciszewskiego 8, 02-786 Warsaw, Poland; jan_slosarz@sggw.edu.pl (J.S.); grzegorz_grodkowski@sggw.edu.pl (G.G.); pawel_solarczyk@sggw.edu.pl (P.S.); piotr_kostusiak@sggw.edu.pl (P.K.); malgorzata_kunowska-slosarz@sggw.edu.pl (M.K.-S.); marek_balcerak@sggw.edu.pl (M.B.); 2 Institute of Mechanical Engineering, Warsaw University of Life Sciences, Nowoursynowska 164, 02-787 Warsaw, Poland; karol_tucki@sggw.edu.pl; 3 Institute of Animal Science and Fisheries, University of Siedlce, ul. B. Prusa 14, 08-110 Siedlce, Poland; krzysztof.mlynek@uws.edu.pl

**Keywords:** body condition score, dairy cows, biomarkers, energy metabolism, oxidative stress, inflammation, mammary gland health, negative energy balance, PLS-DA, early lactation

## Abstract

Body condition score (BCS) is widely used to assess the energy status of dairy cows, yet its relationship with coordinated metabolic and immune responses during early lactation remains incompletely understood. The aim of this study was to characterize the physiological profile associated with low BCS using biomarkers of energy metabolism, hepatic function, oxidative status, inflammation, mammary gland health, milk production, and milk composition. A total of 580 Polish Holstein–Friesian cows in early lactation were classified into four BCS categories and evaluated using univariate and multivariate statistical approaches, including composite physiological indices and partial least squares discriminant analysis (PLS-DA). Cows with the lowest BCS (2.0–2.5) exhibited marked lipid mobilization and hepatic stress, reflected by higher concentrations of non-esterified fatty acids, β-hydroxybutyrate, aspartate aminotransferase, and gamma-glutamyl transferase (all *p* < 0.001). These changes were accompanied by increased oxidative stress, elevated inflammatory activity, alterations in mammary gland biomarkers, and changes in selected milk traits. Composite physiological indices consistently differentiated low-BCS cows from the remaining groups, while PLS-DA identified a distinct biomarker profile associated with low body condition. Among the evaluated variables, interleukin-8 showed the greatest contribution to class discrimination, ranking above conventional metabolic biomarkers in the multivariate analysis. The results indicate that low body condition score is associated with coordinated alterations involving metabolism, liver function, oxidative balance, inflammation, and mammary gland responses rather than isolated metabolic disturbances. The proposed composite physiological indices and the Integrated Low-BCS Phenotype Score (ILBPS) provide an integrative framework for characterizing the physiological phenotype associated with low body condition during early lactation. Prospective longitudinal studies are required to determine whether these biomarkers and composite indices have prognostic value for subsequent metabolic or clinical outcomes.

## 1. Introduction

The transition from late gestation to early lactation is one of the most challenging physiological periods in the productive life of a dairy cow. The onset of lactation is accompanied by a rapid increase in nutrient demand that often exceeds dietary energy intake, resulting in negative energy balance (NEB) [1,2,3,4]. To compensate for this energy deficit, cows mobilize adipose tissue reserves, leading to increased circulating concentrations of non-esterified fatty acids (NEFA) and enhanced hepatic ketogenesis, reflected by elevated β-hydroxybutyrate (BHB) concentrations [1,2,3,4,5]. While these adaptive mechanisms are essential to sustain milk production, excessive or prolonged lipid mobilization can overwhelm metabolic homeostasis and promote metabolic dysfunction, oxidative stress, inflammatory responses, and impaired productive performance [6,7]. Recent evidence further indicates that the transition to lactation involves tightly interconnected metabolic, inflammatory, and oxidative processes rather than independent physiological responses, with disturbances in their coordinated regulation contributing to impaired metabolic health [8].

Body condition score (BCS) has long been used as a practical indicator of body energy reserves and nutritional status in dairy cattle [9,10,11]. However, growing knowledge of metabolic regulation and immunometabolism has broadened its biological significance [12,13,14,15]. Recent studies have further demonstrated that BCS is associated with body weight, circulating biochemical measures, milk production, reproductive performance, and health status, supporting its interpretation within a broader physiological context rather than solely as an estimate of body energy reserves [16,17]. Adipose tissue is now recognized as an active endocrine and immunological organ that secretes adipokines, cytokines, chemokines, and lipid-derived mediators involved in the regulation of energy metabolism, inflammation, and immune function [12,13,14]. Consequently, differences in BCS reflect not only variation in body fat reserves but also differences in physiological adaptation to lactation [9,10,14,15,18]. Cows with excessive body reserve depletion exhibit impaired metabolic flexibility, reduced immune competence, and a greater risk of metabolic and inflammatory disorders, suggesting that low BCS represents a complex physiological state rather than a simple indicator of body condition [4,14,15,18,19].

A central feature of this adaptation is the marked increase in circulating NEFA concentrations resulting from adipose tissue lipolysis [4,18,19,20,21]. Mobilized fatty acids are transported primarily to the liver, where they are oxidized for energy production, converted into ketone bodies, or re-esterified into triglycerides [4,19,21,22]. When the hepatic influx of NEFA exceeds the liver’s metabolic capacity, lipid intermediates accumulate and contribute to mitochondrial dysfunction, endoplasmic reticulum stress, disturbances in glucose metabolism, and hepatocellular injury [5,19,20,22]. These processes are accompanied by increased activities of liver-associated enzymes, particularly aspartate aminotransferase (AST) and gamma-glutamyl transferase (GGT), which are widely used as indicators of hepatic stress during early lactation [5,23,24]. Because the liver coordinates nutrient partitioning, inflammatory signaling, antioxidant defense, and endocrine regulation, hepatic dysfunction is now considered a key component of the transition cow syndrome rather than merely a secondary consequence of negative energy balance [2,5,25,26].

Oxidative stress is now considered one of the key mechanisms linking excessive lipid mobilization with impaired health and productivity in transition dairy cows. Increased oxidation of mobilized fatty acids enhances mitochondrial activity and electron transport, resulting in excessive generation of reactive oxygen species (ROS) [2,27,28,29]. Under physiological conditions, ROS are efficiently neutralized by enzymatic and non-enzymatic antioxidant systems that preserve cellular redox homeostasis. When ROS production exceeds antioxidant capacity, however, oxidative stress develops, leading to lipid peroxidation, protein oxidation, DNA damage, mitochondrial dysfunction, and disruption of intracellular signaling pathways [27,28,29,30,31]. Malondialdehyde (MDA) is widely used as a marker of lipid peroxidation, whereas total antioxidant status (TAS), superoxide dismutase (SOD), glutathione peroxidase (GPx), and glutathione reductase reflect the capacity of antioxidant defense systems [23,30,31,32]. Current evidence further indicates that oxidative stress is not merely a consequence of metabolic overload but also an active regulator of inflammation, immune responses, and tissue remodeling [32,33,34].

The close interaction between metabolism and immunity has led to the development of the concept of immunometabolism, which recognizes nutrient availability, cellular energy status, and immune responses as components of an integrated regulatory network rather than independent physiological processes [12,13,14,18]. In dairy cows, elevated circulating NEFA concentrations activate Toll-like receptor (TLR)-dependent signaling pathways, stimulate nuclear factor κB (NF-κB), and promote the production of pro-inflammatory cytokines, thereby directly coupling metabolic stress with inflammatory activation [14,15,18,20]. Excessive lipid mobilization has also been associated with impaired neutrophil function, reduced phagocytic activity, altered leukocyte metabolism, and increased susceptibility to infectious diseases during the transition period [27,35,36,37,38].

These interactions are further strengthened by oxidative stress. Reactive oxygen species function not only as mediators of cellular injury but also as signaling molecules that activate redox-sensitive pathways, including NF-κB, activator protein-1 (AP-1), and the NLRP3 inflammasome [39,40,41]. Activation of these pathways enhances cytokine production and inflammatory signaling, whereas activated immune cells generate additional ROS, establishing a self-amplifying cycle that perpetuates oxidative damage and inflammation [40,41,42]. This reciprocal relationship between oxidative stress and immune activation is now regarded as one of the principal mechanisms underlying metabolic disorders in transition dairy cows [27,32,42].

The mammary gland is particularly susceptible to these systemic immunometabolic disturbances. Early lactation requires substantial metabolic resources while maintaining effective local immune surveillance, making mammary tissue highly sensitive to alterations in whole-body metabolic status [43,44]. Consequently, metabolic stress has been associated with impaired mammary immune competence and increased susceptibility to intramammary inflammation and mastitis [14,27,43,44,45]. Although somatic cell count (SCC) remains the standard indicator of udder health, it provides only limited information on the molecular events accompanying mammary inflammation [45,46]. This has stimulated growing interest in biomarkers that more directly reflect innate immune activation and epithelial responses.

Among these, interleukin-8 (IL-8) has attracted particular attention because of its central role in neutrophil recruitment and activation during inflammatory responses [47,48]. Increased IL-8 concentrations have been linked to elevated SCC, disruption of mammary epithelial integrity, and activation of innate immune pathways [47,48,49]. Moreover, IL-8 is closely associated with systemic metabolic disturbances, showing strong relationships with NEFA, BHB, AST, and GGT [48]. Other mammary biomarkers, including lactoperoxidase, lysozyme, lactoferrin, and β-lactoglobulin, provide complementary information on innate defense mechanisms, epithelial barrier function, and secretory activity. Their combined assessment offers a broader characterization of mammary health than SCC alone [39,50,51].

Beyond their effects on metabolism and immune function, alterations associated with negative energy balance also influence milk yield and milk composition. Changes in nutrient partitioning during early lactation affect milk protein synthesis, milk fat secretion, and fatty acid metabolism [7,52,53]. In particular, alterations in conjugated linoleic acid (CLA) isomers and oleic acid concentrations have been associated with the intensity of lipid mobilization, suggesting that milk composition may provide additional information on physiological adaptation during early lactation [6,31,49,54,55,56]. However, production-related traits are rarely evaluated together with metabolic, oxidative, hepatic, and inflammatory biomarkers within a single analytical framework, limiting a comprehensive understanding of the biological consequences of excessive body reserve mobilization.

Although considerable progress has been made in understanding individual aspects of transition cow physiology, most previous studies have examined metabolic, oxidative, inflammatory, hepatic, or mammary responses separately. Such reductionist approaches have substantially improved our understanding of individual mechanisms but do not fully reflect the integrated nature of physiological adaptation to early lactation. In reality, metabolic regulation, liver function, redox homeostasis, immune activation, and mammary gland responses interact continuously, forming a coordinated biological network that determines health, productivity, and resilience during the transition period [15,25,26]. Consequently, interpretation based on individual biomarkers alone may underestimate the complexity of physiological adaptation associated with low body condition score.

Recent advances in systems biology have created new opportunities to investigate these complex interactions. Multivariate methods, including partial least squares discriminant analysis (PLS-DA), enable simultaneous evaluation of multiple biologically related variables and facilitate identification of biomarker signatures associated with specific physiological states [57,58]. Likewise, composite physiological indices integrate information from different biological domains into biologically meaningful descriptors of systemic adaptation [25,59]. These approaches are particularly useful for characterizing complex phenotypes in which multiple physiological pathways contribute simultaneously to health status and adaptive capacity.

Despite the widespread use of body condition score in dairy herd management, the integrated biological phenotype associated with low BCS has not been comprehensively characterized. In particular, it remains unclear whether low body condition is accompanied by coordinated alterations in energy metabolism, hepatic function, oxidative balance, inflammatory activation, mammary gland homeostasis, and milk production traits, and which of these biological domains contribute most strongly to the discrimination of physiologically compromised animals.

Although the metabolic, oxidative, inflammatory, and mammary consequences of negative energy balance have been extensively investigated, these physiological domains have often been evaluated separately or using relatively limited biomarker panels. Previous multivariate and composite-biomarker studies have provided important insights into transition-cow physiology; however, simultaneous characterization of lipid mobilization, hepatic stress, redox balance, systemic inflammation, mammary immune responses, and production-related traits within the same animals remains comparatively limited. Consequently, the extent to which these biological domains form a coordinated physiological phenotype associated with low body condition during early lactation remains incompletely characterized. An integrated assessment of these interconnected processes may therefore provide a more comprehensive description of the physiological alterations accompanying excessive body reserve mobilization than evaluation of individual biomarkers or physiological domains alone.

Therefore, the aim of the present study was to characterize the integrated immunometabolic phenotype associated with low body condition score in early-lactation Polish Holstein–Friesian dairy cows. To address this objective, biomarkers related to energy metabolism, hepatic function, oxidative status, innate immunity, mammary gland health, milk production, and milk composition were evaluated simultaneously in cows representing four BCS categories. In addition, composite physiological indices, multivariate statistical analyses, and receiver operating characteristic (ROC) analysis were applied to identify the principal biological pathways underlying the low-BCS phenotype and multivariate statistical analyses and receiver operating characteristic (ROC) analysis were applied to identify the principal biological pathways associated with the low-BCS phenotype and to evaluate the discriminatory performance of individual and integrated biomarker models for distinguishing low-BCS cows from the remaining BCS groups. We hypothesized that cows with the lowest body condition score would exhibit a coordinated immunometabolic phenotype characterized by excessive lipid mobilization, hepatic dysfunction, oxidative imbalance, inflammatory activation, impaired mammary gland homeostasis, and alterations in milk production characteristics.

## 2. Results

Cows were classified into four body condition score (BCS) categories representing distinct levels of body energy reserves: BCS 1 (low BCS; 2.0–2.5), BCS 2 (moderate-low BCS; 2.75–3.0), BCS 3 (moderate BCS; 3.25–3.5), and BCS 4 (high BCS; 3.75–4.0). This classification enabled the evaluation of metabolic, oxidative, inflammatory, mammary gland, and production-related responses across a gradient of body reserve status during early lactation.

Significant differences among BCS classes were detected for most metabolic and oxidative stress biomarkers evaluated (Table 1). The strongest effects of BCS were observed for markers associated with lipid mobilization and hepatic function, including NEFA, GGT, and AST (all *p* < 0.001), which exhibited the highest F-values among the analyzed variables. Significant differences were also found for BHB, MDA, SOD, TAS, glucose, and GluRed (*p* < 0.05). Within the oxidative stress profile, MDA, SOD, and TAS were significantly affected by BCS classification, whereas GPx activity remained unchanged across groups (*p* = 0.485). Notably, biomarkers related to oxidative damage and antioxidant capacity showed stronger responses to BCS than enzymatic antioxidant defense. Overall, the results indicate that BCS classification was primarily associated with alterations in energy metabolism, liver-associated biomarkers, and redox homeostasis, whereas only limited variation was observed for glucose concentrations and no significant differences were detected for GPx activity.

SCC exhibited a strongly right-skewed distribution and substantial heterogeneity of variance on the original scale. Therefore, inferential analysis of SCC was performed after log10 transformation (Table 2). Following transformation, homogeneity of variance was satisfied (Levene’s test, *p* = 0.647), while the effect of BCS category remained highly significant (F = 188.05, *p* < 0.001). Tukey’s HSD post hoc analysis showed that BCS 1 differed significantly from all other BCS categories (*p* < 0.001). BCS 4 also differed significantly from BCS 2 and BCS 3 (*p* = 0.011 and *p* = 0.009, respectively), whereas no difference was detected between BCS 2 and BCS 3 (*p* > 0.05).

Significant differences among BCS classes were observed for most biomarkers associated with mammary gland health and immune response (Table 2). The strongest effect of BCS was detected for IL-8 (F = 488.00, *p* < 0.001), which exhibited the highest discriminatory power among all analyzed variables. Significant differences were also found for SCC, Lp, lysozyme, and β-lactoglobulin (all *p* < 0.001), indicating a pronounced association between BCS classification and inflammatory status. Among the evaluated biomarkers, IL-8, SCC, and Lp showed the largest responses to BCS, as reflected by their high F-values. Lysozyme and β-lactoglobulin were also significantly affected by BCS, although the magnitude of these responses was lower. In contrast, lactoferrin concentrations did not differ among BCS classes (*p* = 0.550), representing the only immune-related parameter unaffected by body condition score. Collectively, the results demonstrate that BCS classification was associated with substantial variation in inflammatory and mammary gland health biomarkers, with the strongest effects observed for indicators of inflammatory activation and udder health status.

Significant differences among BCS classes were observed for milk yield, milk protein content, milk fat content, and CLA c9,t11 concentration (Table 3). Among these variables, milk fat content exhibited the strongest response to BCS classification (F = 32.07, *p* < 0.001), followed by milk yield (F = 6.28, *p* < 0.001), protein content (F = 5.22, *p* = 0.001), and CLA c9,t11 (F = 4.86, *p* = 0.002). In contrast, no significant differences were detected for CLA t10,c12 or C18:1 cis-9 concentrations (*p* > 0.05), indicating that not all components of the fatty acid profile were associated with BCS classification. Overall, production-related traits and selected milk composition parameters showed a stronger response to BCS than the majority of individual fatty acid markers. The observed pattern suggests that body condition score was associated primarily with variation in milk yield and major milk constituents, whereas its relationship with specific fatty acid fractions appeared to be limited.

To assess whether the associations between BCS category and the principal discriminatory biomarkers were influenced by selected cow-level characteristics, additional models were adjusted for parity, exact DIM, and milk yield. BCS category remained strongly associated with IL-8, NEFA, GGT, and AST after adjustment (all *p* < 0.001). The adjusted F-statistics were 512.56 for IL-8, 184.09 for NEFA, 121.40 for GGT, and 100.22 for AST, corresponding to only modest reductions relative to the unadjusted models (2.6%, 4.1%, 2.7%, and 2.7%, respectively). These findings indicate that the associations of BCS category with these principal biomarkers remained robust after accounting for parity, lactation stage, and milk yield (Table 4).

Adjusted models included BCS category and parity as fixed effects and exact DIM and milk yield as covariates. F-statistics refer to the effect of BCS category.

To integrate information obtained from individual biomarkers, composite indices describing major physiological domains affected by body condition score were established (Table 5). The indices summarized alterations related to energy metabolism (EDI), hepatic function (HSI), oxidative balance (OII), inflammatory and mammary gland status (IMI), and milk production traits (MAI). An additional integrated score (ILBPS) was generated to characterize the overall low-BCS phenotype. The biological rationale and direction of change for each index are presented in Table 6.

The composite indices consistently differentiated cows classified as BCS 1 from animals belonging to the remaining BCS categories (Table 6). Across all evaluated physiological domains, BCS 1 cows exhibited positive index values, whereas negative values were observed for BCS 2, BCS 3, and BCS 4. This pattern was evident for the Energy Dysregulation Index (EDI), Hepatic Stress Index (HSI), Oxidative Imbalance Index (OII), Inflammatory–Mammary Index (IMI), and Milk Alteration Index (MAI). Among the evaluated domains, the greatest deviations from the reference phenotype were observed for indices describing metabolic dysregulation, hepatic stress, inflammatory activation, and mammary gland status. Notably, all domain-specific indices converged toward a common profile characterized by elevated values in BCS 1 and consistently reduced values in the remaining groups. A similar trend was observed for the Integrated Low-BCS Phenotype Score (ILBPS), which demonstrated clear separation between BCS 1 and all other BCS classes. The concordant behavior of individual indices indicates that low body condition score was associated with simultaneous alterations in energy metabolism, liver function, redox balance, inflammatory status, and milk-related traits, supporting the existence of a distinct low-BCS molecular phenotype.

Partial least squares discriminant analysis (PLS-DA) demonstrated a clear separation of cows according to body condition score class (Table 7). The strongest discrimination was obtained when cows classified as BCS 1 were contrasted against the combined BCS 2–4 groups. This model achieved the highest classification accuracy, explaining a substantial proportion of the variation associated with class membership and exhibiting good predictive performance. When all four BCS classes were analyzed simultaneously, the discriminatory power of the model decreased, although the classification remained robust. The reduction in model performance suggests a greater overlap among intermediate BCS categories compared with the distinct profile observed for BCS 1 cows. Model stability was further confirmed by cross-validation. The cross-validated model retained high classification accuracy and predictive ability, indicating that the observed separation was not driven by overfitting. Collectively, these findings support the presence of a distinct molecular phenotype associated with low body condition score and demonstrate that the integrated biomarker panel effectively discriminates cows with the lowest BCS from animals with moderate or high body condition.

The PLS-DA-derived VIP analysis identified a limited subset of biomarkers as the principal contributors to discrimination among BCS classes (Table 8). The highest VIP scores were observed for IL-8, NEFA, GGT, SCC, and AST, indicating that inflammatory activation, energy metabolism, liver function, and mammary gland status represented the dominant biological processes underlying group separation. Several additional variables, including Lp, BHB, MDA, TAS, and lysozyme, also exceeded the commonly accepted VIP threshold of 1.0 and therefore contributed meaningfully to model performance. Collectively, these biomarkers reflected interconnected pathways associated with metabolic adaptation, oxidative balance, innate immune response, and inflammatory regulation. In contrast, variables related to antioxidant enzymatic activity (GPx), selected immune proteins (lactoferrin), and fatty acid composition (CLA t10,c12 and C18:1 cis-9) displayed low VIP values and contributed minimally to class discrimination. Similarly, glucose exhibited only a minor contribution despite reaching statistical significance in univariate analyses. Overall, the VIP profile indicated that inflammatory and metabolic biomarkers were the primary determinants of BCS-associated molecular variation, whereas markers related to antioxidant enzymes and selected fatty acid fractions had limited discriminatory value.

ROC analysis demonstrated a high discriminatory performance of biomarkers associated with negative energy balance and inflammatory activation for distinguishing cows with low BCS from cows in the remaining BCS categories. (Table 9). Among the individual biomarkers, IL-8 exhibited the highest discriminatory capacity (AUC = 0.989), followed by NEFA (AUC = 0.973), GGT (AUC = 0.968), AST (AUC = 0.964), and BHB (AUC = 0.910). These findings indicate that inflammatory activation, lipid mobilization, and hepatic dysfunction represent the principal biological processes distinguishing cows with low body condition score. To evaluate whether simultaneous assessment of multiple physiological pathways improves discriminatory performance, integrated logistic models combining inflammatory, metabolic, and hepatic biomarkers were developed. Although all multimarker models achieved excellent discrimination (AUC ≥ 0.989), the combination of IL-8, NEFA, and GGT produced the highest overall discriminatory performance (AUC = 0.990; sensitivity = 100%; specificity = 97.3%). The modest improvement over IL-8 alone suggests that inflammatory signalling is the dominant determinant of the low-BCS phenotype, whereas metabolic and hepatic biomarkers provide complementary rather than independent diagnostic information. Collectively, the ROC analysis independently validates the multivariate PLS-DA results by demonstrating that the low-BCS phenotype is characterized by the concurrent activation of inflammatory, metabolic, and hepatic pathways. The consistently high performance of the integrated models further supports the concept of a coordinated immunometabolic phenotype rather than isolated alterations in single biomarkers.

To evaluate whether the discriminatory performance of the ILBPS was sensitive to the weighting strategy, the original equal-weight score was compared with a VIP-weighted version in an additional sensitivity analysis (Table 10). Both approaches showed identical discriminatory performance based on AUC (0.980). The equal-weight ILBPS achieved a sensitivity of 97.0% and specificity of 97.8%, whereas the VIP-weighted ILBPS achieved a sensitivity of 98.0% and specificity of 97.6%. The AUCs did not differ significantly according to DeLong’s test (*p* = 0.854). These findings indicate that weighting biomarkers according to their PLS-DA-derived VIP values did not materially improve discrimination of cows with low BCS compared with the original equal-weight approach.

Sensitivity analysis comparing the original equal-weight ILBPS with the VIP-weighted ILBPS showed nearly identical discriminatory performance for distinguishing cows classified as BCS 1 from cows in BCS categories 2–4. The equal-weight ILBPS reached an AUC of 0.980, with a sensitivity of 97.0% and specificity of 97.8%, whereas the VIP-weighted ILBPS also reached an AUC of 0.980, with a sensitivity of 98.0% and specificity of 97.6%. Mean score values were similarly distributed across BCS classes for both approaches, with the highest positive values observed in BCS 1 cows and negative values in the remaining BCS groups. Comparison of the correlated ROC curves using DeLong’s test showed no significant difference between the equal-weight and VIP-weighted scores (*p* = 0.854). These findings indicate that weighting biomarkers according to their PLS-DA-derived VIP values did not materially change the discriminatory performance of the ILBPS (Table 10).

The minimal increase in AUC after combining biomarkers indicates that IL-8 alone captures most of the discriminatory information, whereas the remaining biomarkers mainly reinforce biological interpretation rather than substantially improving discriminatory accuracy.

**Figure 1:**
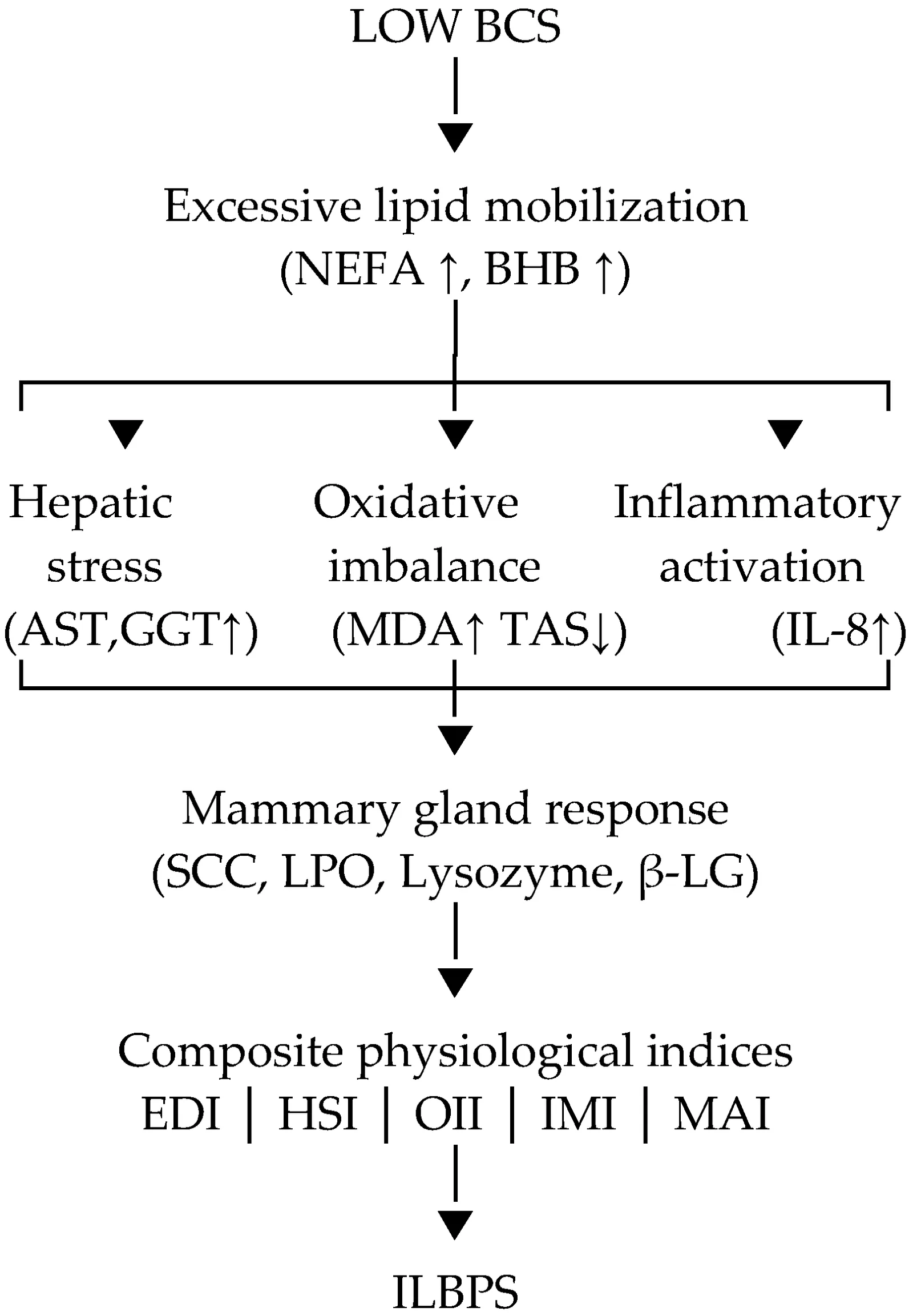
Systems-level representation of the low-BCS immunometabolic phenotype.

To facilitate the integration of the results obtained from univariate, multivariate, and composite index analyses, a systems-level model summarizing the physiological alterations associated with low body condition score was constructed (Figure 1). The model integrates the principal biological domains identified in the present study, including energy metabolism, hepatic function, oxidative balance, inflammatory activation, mammary gland responses, and milk production traits. Excessive body reserve mobilization, reflected by increased circulating NEFA and BHB concentrations, was associated with coordinated alterations in biomarkers of hepatic stress, oxidative imbalance, innate immune activation, and mammary gland homeostasis. These biological domains were subsequently integrated into five composite physiological indices (EDI, HSI, OII, IMI, and MAI), which together formed the Integrated Low-BCS Phenotype Score (ILBPS). The proposed model summarizes the overall pattern of physiological changes observed in cows with low body condition score and provides an integrated representation of the biological relationships identified in the present study.

Pearson correlation analysis revealed a coordinated pattern of associations among metabolic, hepatic, oxidative stress, inflammatory, mammary gland, and milk production biomarkers (Table 11). Markers of negative energy balance, including NEFA and BHB, exhibited strong positive correlations with hepatic enzymes (AST and GGT), oxidative damage (MDA), and inflammatory biomarkers (IL-8 and SCC), indicating a close relationship between metabolic stress and systemic inflammatory activation. Conversely, antioxidant parameters (TAS, SOD, and GPx) were negatively correlated with NEFA, BHB, MDA, IL-8, and SCC, suggesting progressive depletion of antioxidant defense mechanisms under conditions of increased metabolic burden. Among inflammatory markers, IL-8 showed the strongest positive association with SCC (r = 0.82, *p* < 0.001), followed by positive correlations with lactoferrin, lactoperoxidase, and lysozyme, supporting coordinated activation of mammary innate immune responses. Hepatic enzymes AST and GGT were also strongly correlated (r = 0.73, *p* < 0.001), reflecting concurrent hepatic metabolic stress. Milk production traits demonstrated inverse relationships with biomarkers of metabolic and inflammatory stress; milk yield was negatively correlated with NEFA, BHB, IL-8, SCC, and MDA, whereas positive correlations were observed with antioxidant capacity.

Because the pooled correlation structure could partly reflect differences among BCS categories, additional partial correlation analyses controlling for BCS were performed (Table 12). Adjustment for BCS attenuated several associations, indicating that between-group separation contributed to the magnitude of some correlations. Nevertheless, the principal within-system relationships remained evident after adjustment. In particular, IL-8 remained positively associated with SCC (partial r = 0.58), and AST remained strongly associated with GGT (partial r = 0.61). The relationship between the two major indicators of lipid mobilization and ketogenesis, NEFA and BHB, also persisted after adjustment (partial r = 0.52). Significant positive associations were additionally retained between NEFA and GGT (partial r = 0.41), NEFA and AST (partial r = 0.38), and NEFA and IL-8 (partial r = 0.34). These findings indicate that BCS-related group separation contributed to the strength of the pooled correlations but did not fully account for the observed relationships among key metabolic, hepatic, and inflammatory biomarkers.

**Figure 2:**
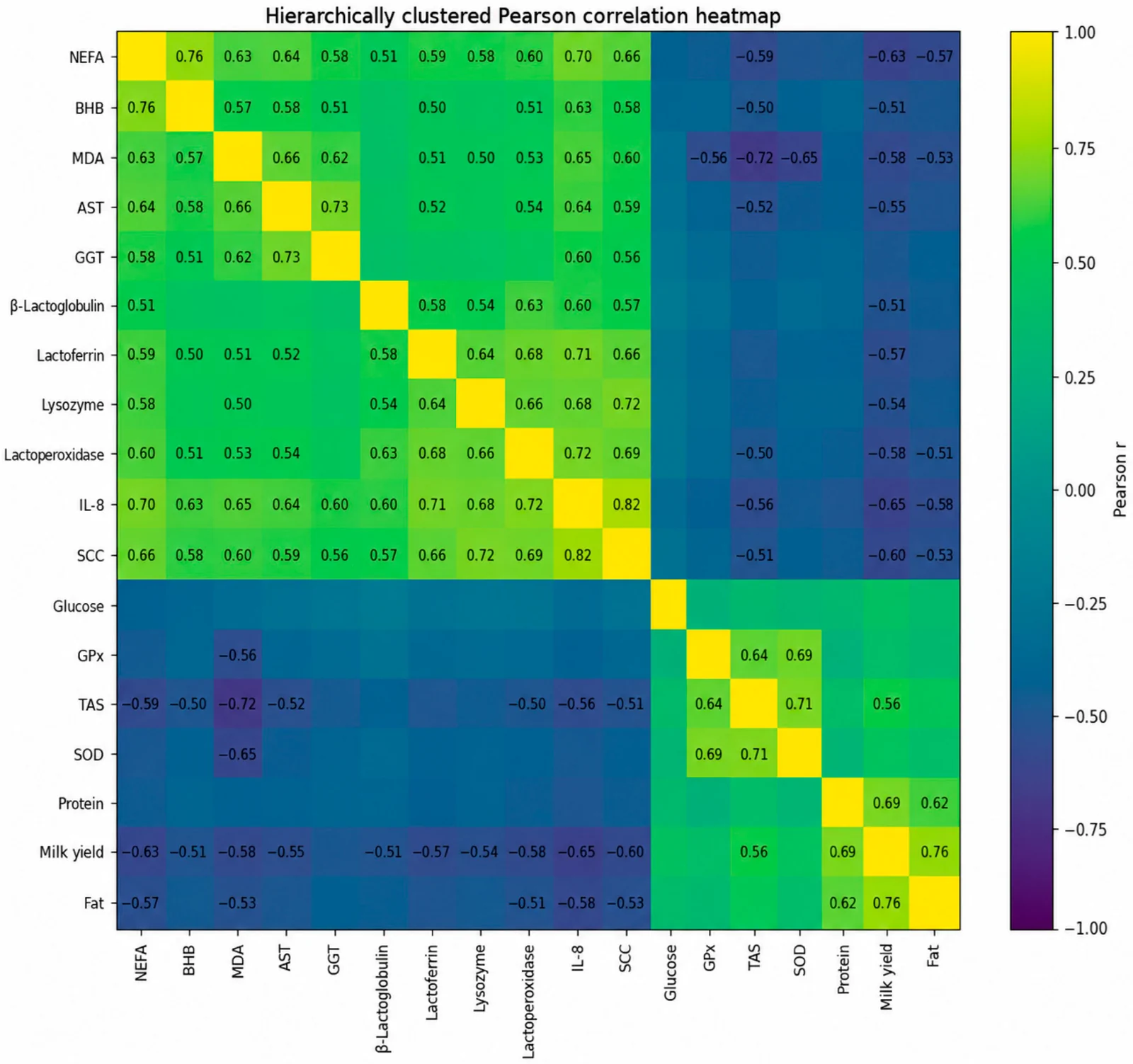
Hierarchically clustered heatmap of Pearson correlations among metabolic, hepatic, oxidative stress, inflammatory, mammary gland, and milk production biomarkers. Cell values represent Pearson correlation coefficients (r). Hierarchical clustering was performed using correlation distance (1 − r) and average linkage.

Hierarchical clustering of the pooled Pearson correlation matrix revealed a structured organization of the biomarker profile, with metabolic and hepatic stress markers clustering closely with inflammatory and mammary-related variables, whereas antioxidant and production-related traits formed a comparatively distinct correlation pattern (Figure 2). Particularly close clustering was observed among IL-8, SCC, and mammary innate-defense biomarkers, while AST and GGT grouped with markers of lipid mobilization and oxidative stress. This visualization complemented the numerical correlation matrix and supported the presence of coordinated, although partially BCS-dependent, relationships among physiological domains.

## 3. Discussion

The present study demonstrates that cows with low body condition score develop a coordinated immunometabolic phenotype that extends well beyond the classical manifestations of negative energy balance. Although BCS has traditionally been regarded as an indicator of body energy reserves, the present findings indicate that low BCS is accompanied by concurrent disturbances in lipid metabolism, hepatic function, oxidative homeostasis, innate immune activation, and mammary gland physiology. Rather than occurring as isolated alterations, these responses formed a coherent biological pattern, as demonstrated by the consistent behavior of individual biomarkers, composite physiological indices, and multivariate analyses. Collectively, these observations support the concept that excessive body reserve depletion is associated with systemic physiological dysregulation rather than a simple reduction in adipose tissue stores.

Importantly, adjustment of the correlation analysis for BCS category attenuated several of the relationships observed in the pooled dataset, indicating that part of the covariance among biomarkers was attributable to physiological differences between BCS groups. However, key associations, particularly IL-8–SCC, AST–GGT, and NEFA–BHB, remained evident after controlling for BCS. Thus, although BCS-related group separation contributed to the overall correlation structure, it did not entirely explain the coordinated relationships observed among inflammatory, hepatic, and metabolic biomarkers.

The metabolic alterations observed in low-BCS cows were among the dominant components of this coordinated response. Increased circulating concentrations of NEFA and BHB are well-established indicators of negative energy balance during early lactation and reflect intensive mobilization of adipose tissue reserves. This interpretation is consistent with recent longitudinal evidence demonstrating close relationships between body-reserve dynamics and hyperketonemia during the first weeks of lactation [60]. However, accumulating evidence suggests that NEFA are not merely alternative energy substrates. Free fatty acids also function as signaling molecules capable of activating innate immune pathways and promoting sterile inflammation. Experimental studies have shown that elevated NEFA concentrations stimulate Toll-like receptor signaling, activate NF-κB and the NLRP3 inflammasome, and increase the production of pro-inflammatory cytokines and chemokines [12,13,14,15,16,17,27]. The parallel increase in metabolic and inflammatory biomarkers observed in the present study is consistent with this mechanism and further supports the existence of a close interaction between lipid mobilization and immune activation during the transition period.

The liver plays a pivotal role in coordinating these physiological adaptations. Mobilized fatty acids are transported to hepatocytes, where they undergo β-oxidation, ketogenesis, or re-esterification. When hepatic metabolic capacity is exceeded, accumulation of lipid intermediates promotes mitochondrial dysfunction, endoplasmic reticulum stress, and impaired hepatocellular function [21,22,25]. The increased AST and GGT activities observed in cows with low BCS are consistent with hepatic adaptation to excessive lipid mobilization and indicate an increased metabolic burden on the liver. Beyond its metabolic functions, the liver has emerged as a central regulator of inflammatory signaling, antioxidant defense, nutrient partitioning, and the acute-phase response during the transition period [25]. Therefore, the hepatic alterations identified in the present study should be interpreted as part of the broader immunometabolic response associated with low body condition score rather than as isolated indicators of liver dysfunction.

One of the most important observations of the present study was the close interplay between metabolic dysregulation, oxidative imbalance, and inflammatory activation in cows with low body condition score. Oxidative stress has long been considered a consequence of excessive metabolic load; however, current evidence indicates that disturbances in redox homeostasis actively influence both metabolic adaptation and inflammatory signaling rather than simply reflecting physiological stress [27,28,31,32]. The simultaneous increase in lipid peroxidation and reduction in antioxidant capacity observed in low-BCS cows indicates that oxidative stress is an integral component of the identified immunometabolic phenotype rather than a secondary consequence of negative energy balance.

This relationship is most likely driven by excessive lipid mobilization. During severe negative energy balance, increased delivery of fatty acids to the liver accelerates mitochondrial β-oxidation, increasing electron transport activity and promoting electron leakage from the respiratory chain, which enhances the generation of reactive oxygen species (ROS) [21,27,28]. Under physiological conditions, antioxidant systems effectively maintain redox balance, but sustained fatty acid oxidation may exceed their protective capacity, resulting in oxidative damage and disruption of cellular homeostasis [31,33]. Similar mechanisms have been described in transition dairy cows experiencing severe metabolic stress, where increased oxidative damage is accompanied by reduced antioxidant capacity, impaired immune function, and greater susceptibility to disease [29,30,34].

Reactive oxygen species should also be viewed as important signaling mediators rather than solely as cytotoxic molecules. By activating redox-sensitive pathways, including NF-κB, AP-1, and the NLRP3 inflammasome, ROS amplify cytokine production and innate immune activation [39,40,42]. The close association between oxidative and inflammatory biomarkers observed in the present study is consistent with this mechanism and supports the concept that oxidative stress links excessive lipid mobilization with activation of the innate immune response. Accordingly, redox imbalance appears to contribute directly to the coordinated immunometabolic disturbances associated with low body condition score.

The different responses of SOD and GPx further illustrate the complexity of antioxidant regulation during early lactation. Increased SOD activity reflects activation of the first enzymatic defense against superoxide radicals, whereas the absence of significant changes in GPx suggests that antioxidant adaptation is selective rather than uniform. Because GPx activity depends largely on selenium availability and glutathione metabolism, its response may remain relatively stable despite increasing oxidative challenge [28,32]. Similar observations have been reported previously, indicating that systemic oxidative stress is better characterized by the combined assessment of oxidative damage and total antioxidant capacity than by individual antioxidant enzymes alone [23,32,33,34]. Overall, the present findings indicate that low body condition score is associated with systemic redox imbalance resulting from inadequate antioxidant compensation during intensive metabolic adaptation. The coordinated changes observed across metabolic, oxidative, and inflammatory biomarkers reinforce the concept of immunometabolic dysregulation during early lactation. Current models of transition cow biology recognize oxidative stress as an active regulator of metabolic adaptation rather than merely a consequence of physiological strain [12,13,27,32]. By amplifying inflammatory signaling, while inflammatory mediators simultaneously promote additional ROS generation, oxidative stress establishes a self-perpetuating cycle that exacerbates metabolic dysfunction [39,40,41,42]. The integrated biomarker profile identified in low-BCS cows is fully consistent with this model and indicates that oxidative imbalance represents a central component of the biological response to excessive body reserve mobilization. Among the evaluated biomarkers, IL-8 showed the greatest ability to discriminate cows with low body condition score, outperforming conventional metabolic indicators such as NEFA, BHB, AST, and GGT. Importantly, inflammatory activation does not appear to be an obligatory feature of a physiologically successful transition to lactation, as relatively limited changes in inflammatory tone have been reported in liver and adipose tissue of cows undergoing a healthy transition [8]. In this context, the pronounced inflammatory alterations observed in low-BCS cows in the present study may reflect stress-related immunometabolic dysregulation rather than a universal physiological response to early lactation [61]. This observation highlights the close relationship between metabolic adaptation and activation of innate immunity during the transition period. Although IL-8 is best known for regulating neutrophil recruitment and activation, it is increasingly recognized as an important mediator linking lipid mobilization with inflammatory signaling through Toll-like receptor- and NF-κB-dependent pathways [12,13,14,15,16,17,18]. Elevated IL-8 concentrations may therefore reflect inflammatory activation associated with metabolic stress; however, the present study does not allow the relative contribution of metabolic and mammary inflammatory processes to circulating IL-8 concentrations to be fully distinguished. The association between IL-8 and mammary immune biomarkers further suggests that these immunometabolic disturbances extend beyond the systemic circulation to the mammary gland. Metabolic stress has been shown to impair leukocyte function, alter epithelial barrier integrity, and modify cytokine production, thereby increasing susceptibility to mammary inflammation [43,44,45,46,47,48,49]. The coordinated increase in IL-8, SCC, lysozyme, lactoperoxidase, and β-lactoglobulin observed in the present study is consistent with activation of innate immune mechanisms within mammary tissue. In contrast, the absence of significant changes in lactoferrin indicates that individual components of the mammary defense system differ in their responsiveness to metabolic stress. Rather than responding uniformly, mammary immune pathways appear to be activated selectively depending on the physiological challenge. These findings support the concept that mammary gland health is closely integrated with whole-body metabolic adaptation and further strengthen the view that low BCS represents a systemic immunometabolic phenotype rather than simply reduced body energy reserves.

Importantly, the strong discriminatory performance of IL-8 should not be interpreted as evidence of specificity for the low-BCS phenotype. Although cows showing overt clinical signs of mastitis or other diseases requiring veterinary intervention were excluded from the study, subclinical mammary inflammation or subclinical metabolic disturbances could not be definitively excluded. The concurrent increases in SCC and mammary innate-defense biomarkers suggest that mammary inflammatory activity may have contributed to the observed IL-8 response, while elevated NEFA and BHB indicate substantial metabolic stress in low-BCS cows. Therefore, potential contributions of subclinical mastitis and ketosis to the IL-8 signal cannot be completely ruled out. Future studies incorporating systematic clinical examination, bacteriological milk testing, disease records, and predefined diagnostic criteria for metabolic disorders will be required to determine the specificity of IL-8 for the low-BCS immunometabolic phenotype.

An important aspect of the present study was the simultaneous integration of biomarkers representing several physiological domains in a relatively large cohort of early-lactation dairy cows. Composite biomarker approaches and multivariate analyses have previously been applied in transition-cow research; therefore, the contribution of the present study lies primarily in the breadth of the biomarker panel and its integrated evaluation across metabolic, hepatic, oxidative, inflammatory, mammary, and production-related domains. The proposed indices—EDI, HSI, OII, IMI, and MAI—were integrated into the Integrated Low-BCS Phenotype Score (ILBPS) to provide a multidimensional description of the physiological alterations associated with low BCS. The high concordance among all domain-specific indices indicates that the physiological alterations associated with low body condition score occur as coordinated responses rather than isolated events. Uniformly elevated EDI, HSI, OII, IMI, and MAI values in BCS 1 cows point to simultaneous disturbances in energy metabolism, hepatic function, oxidative homeostasis, inflammatory activity, and mammary gland physiology. This integrated pattern agrees with current systems biology concepts, which view physiological adaptation as the result of interactions among interconnected biological pathways rather than independent molecular events [57,58,59]. Accordingly, ILBPS may be regarded as an integrative descriptor of the concurrent physiological phenotype associated with low BCS during early lactation. The ability of both the individual indices and the overall ILBPS to consistently distinguish cows with low BCS from the remaining animals further supports the value of composite biomarker approaches for assessing physiological status. From a practical perspective, these indices simplify the interpretation of increasingly complex multidimensional datasets generated in modern dairy production systems. Their integration of information from multiple physiological pathways offers a framework that may provide a basis for future studies evaluating whether integrated biomarker approaches have utility in precision-livestock management. The sensitivity analysis further supported the robustness of the composite scoring approach. Weighting individual biomarkers according to their PLS-DA-derived VIP values did not improve the overall discriminatory performance of the ILBPS, as both the equal-weight and VIP-weighted scores achieved an AUC of 0.980, with no significant difference between the ROC curves (*p* = 0.854). This finding suggests that the ability of the ILBPS to characterize the low-BCS phenotype was not driven primarily by the highest-ranking biomarkers, such as IL-8 or NEFA. Retaining equal weighting therefore preserves the a priori biological contribution of the individual physiological domains while avoiding additional data-driven optimization of the score within the same study population.

The multivariate analyses provided additional evidence for the existence of a distinct biological signature associated with low body condition score. Unlike univariate analyses, PLS-DA evaluates coordinated variation across multiple biomarkers and therefore better reflects the complexity of physiological adaptation [57,58,59]. The markedly stronger separation of BCS 1 cows compared with intermediate BCS classes suggests that metabolic dysregulation is more strongly associated with the lowest BCS category than with intermediate differences across the BCS scale. The VIP analysis further emphasized the dominant contribution of inflammatory activation, lipid mobilization, hepatic stress, and mammary gland responses to discrimination among BCS classes. Conversely, biomarkers such as GPx, lactoferrin, CLA t10,c12, and C18:1 cis-9 contributed little to model performance despite their recognized biological roles. These findings indicate that the low-BCS phenotype is characterized by coordinated disturbances across multiple physiological systems rather than uniform changes in all measured biomarkers.

The present study has several strengths. The relatively large cohort of 580 early-lactation dairy cows provided substantial statistical power for evaluating relationships between body condition score and a broad spectrum of physiological biomarkers. Moreover, the simultaneous assessment of metabolic, oxidative, inflammatory, mammary, and production-related variables enabled a comprehensive characterization of the physiological phenotype associated with low BCS during early lactation. Finally, combining composite physiological indices with multivariate analyses provided a systems-level perspective extending beyond conventional single-biomarker approaches.

Several limitations should also be considered. Because the study was cross-sectional, causal relationships between the observed physiological alterations cannot be established. Moreover, the unequal numbers of cows across BCS categories and potential confounding by cow-level characteristics should be considered when interpreting between-group differences. Although the study population was restricted to multiparous cows in their second and third lactations and to a relatively narrow stage of early lactation, residual variation in parity, exact DIM, and milk yield may have contributed to the observed biomarker patterns. Previous or calving BCS and individual BCS change were not available for analysis; therefore, the direction and magnitude of body-condition change preceding the cross-sectional assessment could not be determined. In addition, individual dry matter intake was not recorded, preventing evaluation of its potential contribution to the observed metabolic differences. In addition, all cows belonged to a single breed and were maintained under similar management conditions, which may limit extrapolation of the findings to other dairy populations. The present investigation also focused on phenotypic biomarkers and did not include transcriptomic, proteomic, or metabolomic analyses that could provide deeper insight into the molecular mechanisms underlying the identified immunometabolic phenotype. Future longitudinal studies integrating multi-omics approaches with physiological monitoring are warranted to validate these observations and determine their temporal relationships and clinical relevance. Furthermore, BCS and all biomarkers were assessed concurrently; therefore, the present study characterizes physiological differences associated with an existing low-BCS phenotype rather than predicting the subsequent development of low BCS or clinical disease. Prospective clinical outcomes, including metabolic disorders, mastitis, culling, and longevity, were not evaluated. Consequently, the prognostic value of individual biomarkers and ILBPS cannot be established from the present data. Longitudinal studies incorporating temporally separated biomarker measurements and clinically relevant outcomes are required to determine whether the identified biomarker patterns have predictive or practical herd-management utility. Furthermore, although animals with overt clinical disease were excluded, the absence of systematic bacteriological milk testing and predefined diagnostic classification of subclinical metabolic disease prevented complete exclusion of subclinical mastitis or ketosis as potential contributors to the observed inflammatory biomarker profile, particularly circulating IL-8.

Overall, the present findings demonstrate that low body condition score is associated with a coordinated immunometabolic phenotype involving interconnected disturbances in metabolic regulation, hepatic function, oxidative balance, inflammatory activity, and mammary gland physiology. These results provide a systems-level framework for characterizing the physiological alterations associated with low body condition during early lactation and provide a basis for future evaluation of integrated biomarker approaches in dairy herd management.

## 4. Materials and Methods

### 4.1. Animals, Housing, and Experimental Design

The study was conducted on 580 multiparous Polish Holstein–Friesian dairy cows maintained at the experimental dairy farm of the Warsaw University of Life Sciences (WULS-SGGW, Warsaw, Poland), Obory 101, 05-520 Konstancin-Jeziorna, Masovian Voivodeship, Poland All cows included in the study were multiparous Polish Holstein–Friesian cows in their second or third lactation and were evaluated between 45 and 65 days in milk (DIM). Body weight was recorded at the time of sampling. Mean body weight was 619 ± 52 kg in cows in their second lactation and 651 ± 51 kg in cows in their third lactation. All animals were in early lactation and sampled between 45 and 65 days in milk (DIM), corresponding to the period of peak milk production and intensive metabolic adaptation.

To provide a more detailed characterization of the study population and to assess the comparability of the BCS categories, group-specific data on parity, exact days in milk (DIM), body weight, and milk yield are presented in Table 13.

Cows were housed in a free-stall system and managed under uniform environmental and nutritional conditions. Animals were fed a total mixed ration (TMR) formulated according to current recommendations for high-producing dairy cows and had ad libitum access to feed and water throughout the study period. Herd management practices, feeding strategy, and housing conditions remained unchanged during the experimental period.

All cows were under continuous veterinary supervision and participated in the routine herd health monitoring program. Animals exhibiting clinical signs of infectious diseases, lameness, reproductive disorders, digestive disturbances, mastitis, or any other condition requiring veterinary intervention at the time of sampling were excluded from the study. No cows received pharmacological treatment at the time of sample collection. Cows showing clinical signs of mastitis at the time of examination were excluded from the study. However, subclinical mastitis was not systematically excluded by bacteriological milk culture or by applying a predefined SCC-based diagnostic threshold. Therefore, the presence of subclinical intramammary inflammation in individual cows, particularly those with elevated SCC, cannot be definitively excluded.

The diets were balanced according to the recommendations of the INRA feeding system. Cows were offered a total mixed ration (TMR) ad libitum and were fed twice daily. The same TMR formulation, adjusted to the production requirements of the herd, was maintained throughout the study period (March–October), thereby avoiding seasonal changes in ration composition. The ingredient composition and available nutritional and production-related characteristics of the TMR are presented in Table 14. Feed intake was monitored based on the amount of TMR offered and the quantity of uneaten feed (orts). Dry matter intake (DMI) was assessed at approximately 3-day intervals throughout the experimental period to monitor feed consumption.

Although the study period extended from March to October, seasonal variation in diet composition was minimized by maintaining the same TMR formulation throughout the entire experimental period. Season itself was not included as an independent experimental factor in the present analysis.

### 4.2. Body Condition Score Classification

Body condition score (BCS) was assessed for all cows by the same trained evaluator using the five-point scoring system described by Edmonson et al. [62], with 0.25-point increments. The scoring procedure was based on visual assessment and palpation of anatomical regions commonly used for evaluating body fat reserves, including the loin, rump, hooks, pins, and tail head. To investigate the relationship between body energy reserves and biological responses during early lactation, cows were classified into four BCS categories. The low-BCS group (BCS 1) included cows with scores ranging from 2.0 to 2.5 (n = 51). The remaining animals were assigned to BCS 2 (2.75–3.0; n = 178), BCS 3 (3.25–3.5; n = 239), and BCS 4 (3.75–4.0; n = 112). The selected classification scheme was designed to represent distinct levels of body reserve mobilization during peak lactation and to facilitate the identification of metabolic, oxidative, inflammatory, and production-related alterations associated with variation in body condition. Subsequent analyses were performed using these BCS categories as the main explanatory factor.

### 4.3. Milk and Blood Sampling

Milk and blood samples were collected from all cows during the same sampling period between 45 and 65 days in milk (DIM). Sample collection was performed as part of the routine herd monitoring program and was carried out in the morning prior to feeding.

Individual milk samples (approximately 250 mL) were collected during routine milking into sterile plastic containers containing a preservative (Mlekostat CC; Polmass, Bydgoszcz, Poland). Immediately after collection, samples were transported under refrigerated conditions to the laboratory of the Warsaw University of Life Sciences for further analyses. Upon arrival, milk samples were divided into aliquots depending on the planned analyses and stored at appropriate temperatures until laboratory determination of milk composition, somatic cell count, inflammatory biomarkers, whey proteins, and fatty acid profile.

Blood samples (10 mL) were collected from the jugular vein using sterile Vacuette^®^ tubes (Greiner Bio-One, Kremsmünster, Austria). Following collection, blood samples were transported to the laboratory and centrifuged at 1800× *g* for 15 min at 4 °C. The obtained serum was carefully separated and stored at −20 °C until analysis. Serum samples were subsequently used for determination of metabolic biomarkers, liver-associated enzymes, and oxidative stress parameters.

To minimize analytical variation, all samples were collected, processed, and analyzed according to standardized laboratory procedures. Milk and blood sampling were performed on the same day for each animal to ensure direct comparison between production, inflammatory, metabolic, and oxidative stress indicators.

### 4.4. Determination of Metabolic Biomarkers

Serum concentrations of glucose, non-esterified fatty acids (NEFA), β-hydroxybutyrate (BHB), aspartate aminotransferase (AST), and gamma-glutamyl transferase (GGT) were determined to evaluate the metabolic and hepatic status of the cows. Blood samples were collected and processed as described above, and serum was stored at −20 °C until analysis. Biochemical analyses were performed using an automatic BS800M biochemical analyzer (PZ Cormay S.A., Warsaw, Poland) according to the manufacturer’s instructions. Commercial diagnostic kits validated for veterinary use were employed for the determination of glucose, AST, and GGT activities. Concentrations of NEFA and BHB were measured using dedicated enzymatic assays routinely applied for the assessment of energy balance and lipid mobilization in dairy cattle. All measurements were performed under standardized laboratory conditions, and internal quality control procedures were applied throughout the analytical period. The selected biomarkers were used to characterize energy metabolism, hepatic function, and the degree of metabolic adaptation during early lactation.

### 4.5. Determination of Oxidative Stress Biomarkers

Oxidative status was evaluated by determining plasma concentrations of malondialdehyde (MDA), total antioxidant status (TAS), glutathione reductase (GluRed), glutathione peroxidase (GPx), and superoxide dismutase (SOD).

Malondialdehyde concentration was determined as an indicator of lipid peroxidation using the thiobarbituric acid reactive substances (TBARS) method. Analyses were performed using a NanoQuant Infinite M200 Pro microplate reader (Tecan Austria GmbH, Grödig, Austria) at a wavelength of 532 nm. Briefly, 250 μL of plasma was mixed with 25 μL of 0.2% 2,6-bis(1,1-dimethylethyl)-4-methylphenol (Sigma-Aldrich, Warsaw, Poland) and 1 mL of 5% trichloroacetic acid (Sigma-Aldrich, Warsaw, Poland). Following centrifugation at 14,000× *g* for 10 min, 750 μL of the supernatant was transferred to a glass tube and mixed with 500 μL of 0.6% thiobarbituric acid (Sigma-Aldrich, Warsaw, Poland). Samples were incubated in a water bath at 90 °C for 45 min, cooled on ice, and centrifuged at 4000× *g* for 5 min. Subsequently, 200 μL of the supernatant was transferred to a microplate and absorbance was measured at 532 nm.

The activities of glutathione reductase (GluRed), glutathione peroxidase (GPx), superoxide dismutase (SOD), and total antioxidant status (TAS) were determined using a NanoQuant Infinite M200 Pro microplate reader (Tecan Austria GmbH, Grödig, Austria) and commercial Randox assay kits (Randox Laboratories Ltd., Crumlin, UK), according to the manufacturer’s instructions. Glutathione reductase activity was measured using the Glutathione Reductase assay kit (Cat. No. GR2608; Randox Laboratories Ltd., Crumlin, UK), glutathione peroxidase activity using the Ransel kit (Cat. No. RS505; Randox Laboratories Ltd., Crumlin, UK), superoxide dismutase activity using the Ransod kit (Cat. No. SD126; Randox Laboratories Ltd., Crumlin, UK), and total antioxidant status using the TAS kit (Cat. No. NX2331; Randox Laboratories Ltd., Crumlin, UK).

Total antioxidant status was determined based on the suppression of ABTS^®^ radical cation formation by antioxidants present in the sample. The incubation of ABTS^®^ with metmyoglobin and hydrogen peroxide generates the ABTS^®^+ radical, which produces a characteristic blue-green color measured at 600 nm. The degree of color suppression is proportional to the antioxidant capacity of the sample.

Superoxide dismutase activity was quantified based on the inhibition of superoxide radical formation generated by the xanthine–xanthine oxidase system. Glutathione peroxidase activity was determined from the rate of glutathione oxidation in the presence of cumene hydroperoxide, whereas glutathione reductase activity was measured through the NADPH-dependent reduction of oxidized glutathione (GSSG) to reduced glutathione (GSH). All analyses were performed in duplicate under standardized laboratory conditions.

### 4.6. Determination of Inflammatory and Mammary Gland Biomarkers

Inflammatory status and mammary gland health were evaluated using somatic cell count (SCC), interleukin-8 (IL-8), lactoferrin (LF), lysozyme (LZ), lactoperoxidase (Lp), and β-lactoglobulin (BLG).

Somatic cell count was determined using a Somacount 150 analyzer (Bentley Instruments Inc., Chaska, MN, USA). Prior to biochemical analyses, milk samples were centrifuged at 5000× *g* for 15 min to remove the fat layer. The resulting skimmed fraction was subsequently used for the determination of whey proteins and cytokines.

For whey protein analysis, milk samples were acidified using a 10% acetic acid solution to precipitate casein. Following centrifugation at 14,000× *g* for 15 min, the supernatant was filtered through a nylon membrane filter and used for chromatographic analyses. Concentrations of lactoferrin, lysozyme, lactoperoxidase, and β-lactoglobulin were determined by reversed-phase high-performance liquid chromatography (RP-HPLC) using an Agilent 1100 Series chromatographic system (Agilent Technologies, Waldbronn, Germany) equipped with a Jupiter C18 300 Å column (Phenomenex, Torrance, CA, USA). The mobile phases consisted of acetonitrile, ultrapure water, and trifluoroacetic acid, and protein separation was performed using a gradient elution program. Detection was carried out at 220 nm. Identification of individual proteins was confirmed using certified reference standards (Sigma-Aldrich, St. Louis, MO, USA). All samples were analyzed in duplicate.

The concentration of IL-8 in milk was determined using a bovine-specific enzyme-linked immunosorbent assay (ELISA) kit (MyBioSource Inc., San Diego, CA, USA) according to the manufacturer’s protocol. Briefly, standards and samples were added to microplate wells pre-coated with monoclonal antibodies specific for bovine IL-8 and incubated to allow antigen binding. After washing, enzyme-conjugated detection antibodies were added, followed by substrate solution. Absorbance was measured using a microplate reader, and IL-8 concentrations were calculated from a standard calibration curve generated for each assay. All samples and standards were analyzed in duplicate.

### 4.7. Milk Composition and Fatty Acid Analysis

Milk yield was recorded on the day of sample collection as part of the routine herd performance monitoring system. Milk composition, including fat and protein content, was determined using a MilkoScan FT-120 analyzer (FOSS Electric A/S, Hillerød, Denmark). Analyses were performed according to the manufacturer’s instructions and routinely validated laboratory procedures.

Fatty acid analysis was performed on milk fat samples following methyl ester preparation according to the transesterification method described in PN-EN ISO 5509:2000 [63]. Fatty acid methyl esters (FAME) were separated and quantified using an Agilent 7890 gas chromatograph (Agilent Technologies, Waldbronn, Germany) equipped with a Varian Select FAME capillary column. Chromatographic separation was carried out under the following temperature program: 130 °C for 1 min; 130–170 °C at 6.5 °C min^−1^; 170–215 °C at 2.75 °C min^−1^; 215 °C for 12 min; 215–230 °C at 20 °C min^−1^; and 230 °C for 3 min. Helium was used as the carrier gas at a constant flow rate of 25 cm s^−1^. The injector and detector temperatures were maintained at 240 °C and 300 °C, respectively. Individual fatty acids were identified by comparison of retention times with certified methyl ester standards (Supelco, Bellefonte, PA, USA). Quantitative analyses were performed in duplicate for each sample. Particular attention was given to conjugated linoleic acid (CLA) isomers, including cis-9, trans-11 CLA (CLA c9,t11) and trans-10, cis-12 CLA (CLA t10,c12), as well as oleic acid (C18:1 cis-9), due to their recognized biological significance and potential associations with metabolic and inflammatory status in dairy cows.

### 4.8. Construction of Composite Molecular Phenotype Indices

To obtain an integrated assessment of the complex physiological alterations associated with body condition score (BCS), composite indices were developed to summarize biologically related biomarkers into functionally interpretable domains. Because individual biomarkers represent only selected aspects of metabolic adaptation, the construction of composite indices was intended to reduce biological complexity while preserving information derived from multiple interconnected physiological pathways.

Prior to index construction, all continuous variables were standardized using z-score transformation:

Z_i_ = (X_i_ − µ)/σ

where *x* represents the observed value, *μ* is the population mean, and *σ* is the corresponding standard deviation.

Standardization was performed to eliminate differences arising from measurement units and biological scales, thereby ensuring that each biomarker contributed equally to the corresponding composite index. Because the measured biomarkers differed in their biological interpretation, all variables were oriented before aggregation so that increasing index values consistently represented deterioration of physiological status. Biomarkers positively associated with metabolic dysfunction (NEFA, BHB, AST, GGT, MDA, IL-8, SCC, lysozyme, lactoperoxidase and β-lactoglobulin) retained their original orientation, whereas biomarkers reflecting physiological protection or improved homeostasis (TAS, milk yield, milk fat content and CLA c9,t11) were reverse-coded prior to index calculation.

The composite indices used in the present study were developed specifically for this analysis and were not adopted from previously published scoring systems. The selection and grouping of individual biomarkers into physiological domains were based on their established biological roles in energy metabolism, hepatic function, oxidative balance, inflammatory and mammary responses, and milk production, as supported by previous studies.

The Energy Dysregulation Index (EDI) was calculated from standardized NEFA and BHB values because both biomarkers reflect the intensity of lipid mobilization and negative energy balance during early lactation. Previous work has established the usefulness of NEFA and BHB for characterizing metabolic status and ketosis-related alterations in dairy cows, including our earlier studies on metabolic profiles and postpartum energy imbalance [10,11,55]. The 2016 review specifically identified NEFA and β-hydroxybutyric acid as key components of metabolic profiling in dairy cows, while the 2019 study classified postpartum cows using BHBA and NEFA concentrations and demonstrated their association with ketosis. More recently, BCS restoration, circulating fatty acids, and BHBA were shown to track the metabolic burden associated with negative energy balance during lactation.

The Hepatic Stress Index (HSI) combined GGT and AST activities as indicators of hepatic and metabolic burden. Their selection was supported by our previous study demonstrating significant alterations in AST and GGT activities in dairy cows differing in metabolic status during early lactation [48]. In that study, ketotic cows exhibited the highest GGT activity, whereas AST activity differed markedly according to metabolic status, supporting their complementary use in characterizing hepatic metabolic stress.

The Oxidative Imbalance Index (OII) incorporated MDA, SOD, and TAS to represent complementary aspects of oxidative status, including lipid peroxidation and antioxidant defence. The biological rationale for combining these biomarkers was based on previous work describing oxidative stress as an imbalance between pro-oxidant processes and antioxidant defence systems [64], as well as our experimental findings showing alterations in MDA, SOD, and TAS in dairy cows with different metabolic status [48]. In the 2022 study, MDA, SOD, and TAS were evaluated concurrently, and significant relationships between BHBA and oxidative-stress markers were demonstrated.

The Inflammatory–Mammary Index (IMI) integrated IL-8, SCC, lysozyme, lactoperoxidase, and β-lactoglobulin to represent coordinated inflammatory activation and mammary gland response. This biomarker combination was supported by our previous work demonstrating strong associations between circulating IL-8 and SCC, as well as significant relationships between IL-8 and mammary innate-defence proteins, including lysozyme, lactoperoxidase, and β-lactoglobulin [48]. Specifically, IL-8 was significantly correlated with lysozyme, β-lactoglobulin, and lactoperoxidase, supporting their joint interpretation as indicators of mammary inflammatory activity.

The Milk Alteration Index (MAI) integrated milk yield, fat content, protein content, and CLA c9,t11 concentration because these variables reflect production and compositional responses to changes in energy status and lipid mobilization. Previous studies have shown that negative energy balance, body condition loss, and elevated BHBA are associated with alterations in milk production and milk fat composition [10,55]. In postpartum Holstein–Friesian cows, elevated BHBA was associated with marked changes in milk fat and CLA concentrations, including reduced CLA c9,t11 in cows with higher BHBA. More recent work demonstrated relationships among BCS, BHBA, milk production, and milk fat characteristics during lactation.

For each physiological domain, the corresponding composite index was calculated as the arithmetic mean of the standardized and directionally oriented variables assigned to that domain. Finally, the Integrated Low-BCS Phenotype Score (ILBPS) was calculated from EDI, HSI, OII, IMI, and MAI to provide an overall integrative descriptor of the concurrent metabolic, hepatic, oxidative, inflammatory, mammary, and production-related alterations associated with low body condition score. Because ILBPS and the five domain-specific indices were developed specifically for the present study, no direct literature references exist for the indices themselves; the cited literature provides the biological rationale for the selection and grouping of their individual components.

Consequently, positive values of every composite index indicate greater biological disturbance, whereas negative values correspond to more favorable physiological status.

Five biologically defined domain-specific indices were subsequently generated:

— Energy Dysregulation Index (EDI) summarizing lipid mobilization and negative energy balance (NEFA and BHB);
— Hepatic Stress Index (HSI) describing hepatocellular metabolic burden (AST and GGT);
— Oxidative Imbalance Index (OII) integrating oxidative damage and antioxidant capacity (MDA, SOD and reverse-coded TAS);
— Inflammatory–Mammary Index (IMI) reflecting systemic inflammatory activation and mammary immune response (IL-8, SCC, lysozyme, lactoperoxidase and β-lactoglobulin);
— Milk Alteration Index (MAI) representing production-related alterations associated with metabolic status (milk yield, fat percentage, protein percentage and CLA c9,t11).

Each domain-specific index was calculated as the arithmetic mean of the standardized biomarkers assigned to the corresponding biological pathway.

Finally, an Integrated Low-BCS Phenotype Score (ILBPS) was calculated as the arithmetic mean of the five domain-specific indices:

ILBPS = (EDI + HSI + OII + IMI + MAI)/5

The ILBPS was designed to provide an overall quantitative descriptor of the coordinated physiological alterations associated with low body condition score by integrating metabolic, hepatic, oxidative, inflammatory and production-related responses into a single composite measure. Equal weighting of biomarkers within each index was intentionally adopted because no experimental or biological evidence currently exists to justify differential weighting of individual variables. This approach is commonly applied in exploratory systems biology studies when composite scores are constructed from biomarkers representing complementary physiological processes. Furthermore, z-score normalization ensured that biomarkers with larger numerical ranges did not dominate the final indices. The biological composition of all indices was defined a priori, based on current knowledge of transition cow physiology and immunometabolism, rather than being derived statistically from the present dataset. Consequently, the indices should be interpreted as biologically informed integrative descriptors rather than latent statistical factors.

As a sensitivity analysis, a VIP-weighted version of the Integrated Low-BCS Phenotype Score was additionally calculated to evaluate the robustness of the equal-weight scoring strategy. Within each physiological domain, directionally oriented standardized biomarkers were weighted according to their Variable Importance in Projection (VIP) values derived from the PLS-DA model. Each weighted domain score was calculated as the sum of the products of the standardized biomarker values and their corresponding VIP weights divided by the sum of VIP weights within that domain. The resulting VIP-weighted domain scores were subsequently averaged with equal weighting across the five physiological domains to obtain the VIP-weighted ILBPS. This analysis was performed as a sensitivity analysis rather than as a replacement for the primary equal-weight ILBPS, because VIP weights are data-driven and were derived from the same study population.

### 4.9. Multivariate Analysis (PLS-DA and VIP)

To evaluate the integrated biological differences among body condition score (BCS) classes and identify the biomarkers contributing most strongly to group discrimination, partial least squares discriminant analysis (PLS-DA) was performed. The analysis included all metabolic, oxidative stress, inflammatory, mammary gland, and milk production variables measured in the study. Prior to multivariate analysis, all variables were standardized using z-score normalization to eliminate differences arising from measurement scales and units. Body condition score class was used as the categorical response variable, whereas the measured biomarkers constituted the predictor matrix. PLS-DA models were constructed to assess both the discrimination among all four BCS classes and the separation of cows with low body condition score (BCS 1) from cows classified as BCS 2–4. Model performance was evaluated using classification accuracy, the proportion of explained variance (R^2^Y), and predictive ability (Q^2^). Model robustness was assessed using repeated cross-validation procedures and permutation testing (1000 permutations) implemented in MetaboAnalyst 6.0. The original model was considered valid when the observed R^2^Y and Q^2^ values exceeded those obtained from randomly permuted class labels. The relative contribution of individual biomarkers to model performance was determined using Variable Importance in Projection (VIP) scores. Variables with VIP values greater than 1.0 were considered important contributors to class discrimination, whereas biomarkers with VIP values below 0.8 were considered to have limited discriminatory value. Biomarkers were subsequently ranked according to their VIP scores to identify the principal biological pathways associated with variation in body condition score. To facilitate biological interpretation, VIP-ranked variables were grouped into functional categories related to energy metabolism, hepatic function, oxidative stress, inflammatory response, mammary gland health, and milk composition. The resulting multivariate models were used to characterize the integrated molecular phenotype associated with low body condition score in dairy cows. Model performance was evaluated using classification accuracy, explained variance (R^2^Y), and predictive ability (Q^2^). Model robustness was assessed using repeated cross-validation procedures implemented in MetaboAnalyst 6.0. To further evaluate model validity and minimize the risk of overfitting, permutation testing (1000 permutations) was performed. The significance of the PLS-DA models was confirmed when the original R^2^Y and Q^2^ values exceeded those obtained from the permuted models.

### 4.10. Statistical Analysis

Statistical analyses were performed using IBM SPSS Statistics software (version 29.0; IBM Corp., Armonk, NY, USA) and MetaboAnalyst 6.0 (McGill University, Montreal, QC, Canada). Prior to statistical evaluation, all variables were screened for missing values, visually inspected for potential outliers, and tested for distributional assumptions. Normality was assessed using the Shapiro–Wilk test together with visual inspection of Q–Q plots, whereas homogeneity of variances among body condition score (BCS) groups was evaluated using Levene’s test. No missing observations requiring data imputation were identified. Descriptive statistics were calculated for all variables and are presented as mean ± standard deviation (SD).

Differences among BCS classes were evaluated using one-way analysis of variance (ANOVA), with BCS category included as the fixed effect. When a significant overall effect was detected, pairwise comparisons among groups were performed using Tukey’s honestly significant difference (HSD) post hoc test to control the family-wise error rate. All statistical tests were two-sided, and statistical significance was declared at *p* < 0.05. To assess the potential influence of cow-level confounding variables, additional adjusted models were fitted for the principal discriminatory biomarkers (IL-8, NEFA, GGT, and AST). BCS category and parity were included as fixed effects, whereas exact days in milk (DIM) and milk yield were included as covariates. Adjusted F-statistics for the BCS effect were compared with those obtained from the corresponding unadjusted models. Statistical significance was declared at *p* < 0.05.

Because SCC showed a strongly right-skewed distribution and marked heterogeneity of variance on the original scale, SCC values were log10-transformed prior to parametric statistical analysis. Normality and homogeneity of variance were reassessed after transformation using the Shapiro–Wilk and Levene tests, respectively. Group differences in log10-transformed SCC were subsequently evaluated using one-way ANOVA followed by Tukey’s HSD post hoc test. For ease of biological interpretation, SCC values are presented in Table 2 on the original scale as arithmetic mean ± SD, whereas statistical inference was based on log10-transformed values.

Associations among metabolic, oxidative stress, inflammatory, mammary gland, and milk production biomarkers were quantified using Pearson product–moment correlation coefficients (*r*). The statistical significance of correlations was assessed using two-sided significance tests. Correlation matrices were subsequently visualized as hierarchical clustered heatmaps to facilitate identification of biologically meaningful relationships among biomarkers and to provide an integrated overview of the interactions among physiological pathways. Pearson correlation coefficients were initially calculated using the pooled dataset to characterize the overall relationships among metabolic, hepatic, oxidative, inflammatory, mammary, and production-related biomarkers. Because several biomarkers differed substantially among BCS categories, additional partial correlation analyses controlling for BCS category were performed to distinguish associations attributable primarily to between-group separation from relationships persisting independently of BCS classification. In addition, within-group Pearson correlations were examined separately for each BCS category as a sensitivity analysis to assess the consistency and direction of the principal biomarker relationships within BCS strata. The pooled Pearson correlation matrix was additionally visualized as a hierarchically clustered heatmap. Hierarchical clustering was performed using correlation distance (1 − r) as the distance metric and average linkage as the agglomeration method. The resulting clustering structure was used to visualize groups of biomarkers exhibiting similar correlation profiles across physiological domains.

To obtain a systems-level representation of the physiological alterations associated with body condition score, composite molecular phenotype indices were calculated as described in Section 4.8. Individual index values were calculated for each animal following z-score standardization of the constituent biomarkers and subsequent aggregation into biologically defined domains. Differences in index values among BCS groups were evaluated using one-way ANOVA followed by Tukey’s HSD test.

Multivariate analyses were performed using both unsupervised and supervised approaches. Principal component analysis (PCA) was initially conducted to explore the overall structure of the dataset, evaluate natural clustering of cows according to BCS class, detect potential multivariate outliers, and visualize the principal sources of biological variation. Prior to PCA, all variables were mean-centered and autoscaled (unit variance scaling; z-score transformation) to ensure equal weighting of biomarkers measured on different numerical scales.

Subsequently, partial least squares discriminant analysis (PLS-DA) was performed to evaluate the discriminatory capacity of the integrated biomarker panel and to characterize molecular phenotypes associated with variation in body condition score. Separate models were generated for (i) discrimination among all four BCS classes and (ii) binary classification of cows with low body condition score (BCS 1) versus the remaining animals (BCS 2–4). The optimal number of latent variables was selected using internal cross-validation procedures implemented in MetaboAnalyst. Model performance was assessed using classification accuracy, explained variance of the response variable (R^2^Y), and predictive ability (Q^2^). To evaluate model robustness and minimize the risk of overfitting, repeated cross-validation procedures were applied, and statistical significance was further verified using permutation testing based on 1000 random permutations of class labels. PLS-DA models were considered robust when the original R^2^Y and Q^2^ values exceeded those obtained from the permuted datasets.

The relative contribution of individual biomarkers to class discrimination was assessed using Variable Importance in Projection (VIP) scores. Biomarkers with VIP values greater than 1.0 were considered major contributors to discrimination among BCS classes, whereas variables with VIP values below 0.8 were regarded as having limited discriminatory importance. Variables were subsequently ranked according to their VIP values to identify the principal metabolic, hepatic, oxidative, inflammatory, mammary gland, and production-related pathways underlying the low-BCS molecular phenotype.

Receiver operating characteristic (ROC) curve analysis was performed to evaluate the discriminatory performance of individual biomarkers and integrated biomarker panels for distinguishing cows classified as BCS 1 from animals belonging to BCS categories 2–4. Discriminatory performance was quantified by calculating the area under the ROC curve (AUC) together with 95% confidence intervals (95% CI), sensitivity, specificity, and optimal cut-off values determined using the Youden index. To evaluate whether simultaneous assessment of biomarkers improved discriminatory performance, integrated biomarker models were constructed using binary logistic regression based on combinations of the highest-ranking biomarkers identified by the PLS-DA and VIP analyses. The discriminatory performance of these multivariable models was subsequently evaluated using ROC analysis and compared with that of individual biomarkers. The discriminatory performance of the equal-weight and VIP-weighted ILBPS was compared as a sensitivity analysis. ROC curves were generated for both scores for discrimination of BCS 1 from BCS 2–4 cows, and differences between the corresponding AUCs were evaluated using DeLong’s test for correlated ROC curves.

All statistical analyses were performed using two-sided tests, and differences were considered statistically significant at *p* < 0.05.

## 5. Conclusions

The present study demonstrates that low body condition score represents a distinct immunometabolic phenotype rather than merely a reduction in body energy reserves during early lactation. Cows with low BCS exhibited coordinated alterations in energy metabolism, hepatic function, oxidative homeostasis, inflammatory activity, mammary gland immunity, and milk production traits, indicating that excessive body reserve mobilization is associated with a complex systems-level physiological response. The close integration of metabolic, oxidative, inflammatory, and mammary biomarkers, together with the consistent behavior of the composite physiological indices and multivariate analyses, supports a coordinated pattern of physiological alterations associated with the low-BCS phenotype. Among the evaluated biomarkers, IL-8 emerged as the most informative indicator of this integrated physiological state, highlighting the prominent association of inflammatory activation with the low-BCS phenotype. Rather than occurring as isolated alterations, lipid mobilization, oxidative stress, hepatic stress, and innate immune activation were concurrently associated with the low-BCS immunometabolic phenotype. The composite physiological indices and the Integrated Low-BCS Phenotype Score proposed in this study provide an integrative approach for combining information from multiple biological domains into a biologically interpretable assessment of physiological status. This approach complements conventional single-biomarker evaluation by providing an integrated characterization of the concurrent physiological phenotype associated with low BCS. Overall, these findings advance the current understanding of the physiological characteristics associated with low body condition during early lactation by demonstrating that low BCS is characterized by coordinated immunometabolic dysregulation rather than isolated metabolic disturbances. Future longitudinal studies integrating transcriptomic, proteomic, metabolomic, physiological, and clinically relevant outcome data will be essential to validate these associations, elucidate the molecular mechanisms underlying the response to negative energy balance, and determine whether the identified biomarker patterns and composite indices have prognostic or practical utility in precision dairy management.

## Figures and Tables

**Table 1:** Metabolic status and oxidative stress biomarkers in dairy cows classified according to body condition score (BCS). Data are presented as mean ± SD. Differences among groups were evaluated using one-way ANOVA.

Variable	BCS 1 (n = 51)	BCS 2 (n = 178)	BCS 3 (n = 239)	BCS 4 (n = 112)	F	*p*
Glucose (mg/dL)	63.68 ± 5.53 ^a^	66.02 ± 7.51 ^a^	65.90 ± 8.66 ^a^	63.64 ± 6.52 ^a^	3.50	0.015
NEFA (mmol/L)	1.41 ± 0.59 ^a^	0.35 ± 0.32 ^b^	0.37 ± 0.27 ^b^	0.38 ± 0.30 ^b^	155.77	<0.001
BHB (mmol/L)	1.19 ± 0.46 ^a^	0.74 ± 0.26 ^b^	0.78 ± 0.33 ^b^	0.81 ± 0.29 ^b^	27.97	<0.001
GGT (U/L)	144.20 ± 49.13 ^b^	41.30 ± 42.18 ^b^	36.62 ± 36.17 ^b^	30.30 ± 24.46 ^b^	128.47	<0.001
AST (U/L)	140.94 ± 36.23 ^b^	83.87 ± 22.07 ^b^	86.29 ± 18.40 ^b^	87.74 ± 18.26 ^b^	101.68	<0.001
MDA (µmol/L)	38.90 ± 12.98 ^a^	30.07 ± 8.46 ^b^	29.12 ± 7.50 ^b^	28.97 ± 6.33 ^b^	21.23	<0.001
GluRed (U/L)	95.61 ± 42.09 ^a^	88.83 ± 35.63 ^ab^	80.42 ± 38.16 ^b^	81.54 ± 34.16 ^ab^	3.55	0.014
GPx (U/L)	471.02 ± 187.15 ^a^	490.11 ± 209.96 ^a^	465.88 ± 202.21 ^a^	454.20 ± 209.56 ^a^	0.82	0.485
SOD (U/L)	288.25 ± 121.86 ^a^	237.36 ± 75.53 ^b^	246.72 ± 73.22 ^b^	237.91 ± 62.75 ^b^	6.19	<0.001
TAS (mmol/L)	0.83 ± 0.85 ^a^	1.77 ± 1.16 ^b^	1.81 ± 1.29 ^b^	1.49 ± 1.25 ^b^	10.62	<0.001

BCS 1 (low BCS; 2.0–2.5), BCS 2 (moderate-low BCS; 2.75–3.0), BCS 3 (moderate BCS; 3.25–3.5), and BCS 4 (high BCS; 3.75–4.0). BCS, body condition score; NEFA, non-esterified fatty acids; BHB, β-hydroxybutyrate; GGT, gamma-glutamyl transferase; AST, aspartate aminotransferase; MDA, malondialdehyde; GluRed, glutathione reductase; GPx, glutathione peroxidase; SOD, superoxide dismutase; TAS, total antioxidant status. Values are presented as mean ± standard deviation (SD). Differences among groups were evaluated using one-way analysis of variance (ANOVA). Statistical significance was declared at *p* < 0.05. Within each row, means not sharing a common superscript letter differ significantly according to Tukey’s HSD post hoc test (*p* < 0.05).

**Table 2:** Mammary gland inflammation and immune response biomarkers.

Variable	BCS 1	BCS 2	BCS 3	BCS 4	F	*p*
SCC (×10^3^ cells/mL)	1384.49 ± 1765.33 ^a^	85.85 ± 93.96 ^b^	78.62 ± 80.11 ^b^	60.87 ± 67.06 ^c^	188.05	<0.001
IL-8 (ng/mL)	10.87 ± 5.99 ^a^	0.82 ± 0.39 ^b^	0.78 ± 0.33 ^b^	0.74 ± 0.36 ^b^	488.00	<0.001
Lysozyme (µg/L)	38.67 ± 15.64 ^a^	19.68 ± 11.29 ^b^	18.70 ± 15.62 ^b^	18.16 ± 8.90 ^b^	34.63	<0.001
Lactoferrin (g/L)	0.255 ± 0.164 ^a^	0.236 ± 0.138 ^a^	0.246 ± 0.256 ^a^	0.218 ± 0.106 ^a^	0.70	0.550
β-Lactoglobulin (g/L)	4.69 ± 1.27 ^a^	3.18 ± 1.09 ^b^	3.19 ± 1.09 ^b^	3.26 ± 1.16 ^b^	27.51	<0.001
Lp (mg/L)	0.669 ± 0.513 ^a^	0.277 ± 0.126 ^b^	0.279 ± 0.152 ^b^	0.250 ± 0.111 ^b^	62.03	<0.001

BCS 1 (low BCS; 2.0–2.5), BCS 2 (moderate-low BCS; 2.75–3.0), BCS 3 (moderate BCS; 3.25–3.5), and BCS 4 (high BCS; 3.75–4.0). BCS, body condition score; SCC, somatic cell count; IL-8, interleukin-8; Lp, lactoperoxidase. Values are presented as mean ± standard deviation (SD). Differences among groups were evaluated using one-way analysis of variance (ANOVA). Statistical significance was declared at *p* < 0.05. Within each row, means not sharing a common superscript letter differ significantly according to Tukey’s HSD post hoc test (*p* < 0.05). Values are presented as arithmetic mean ± SD on the original scale. SCC was log10-transformed prior to statistical analysis because of right-skewness and heterogeneity of variance; the F- and *p*-values reported for SCC are based on the log10-transformed data. For all other variables, differences among BCS categories were evaluated using one-way ANOVA. Within each row, means not sharing a common superscript letter differ significantly according to Tukey’s HSD post hoc test (*p* < 0.05).

**Table 3:** Milk production traits and selected fatty acid profile characteristics.

Variable	BCS 1	BCS 2	BCS 3	BCS 4	F	*p*
Milk yield (kg/day)	28.54 ± 7.74 ^a^	34.01 ± 7.14 ^b^	32.42 ± 8.41 ^b^	33.19 ± 8.95 ^b^	6.28	<0.001
Protein (%)	3.61 ± 0.41 ^a^	3.38 ± 0.33 ^b^	3.43 ± 0.39 ^b^	3.39 ± 0.39 ^b^	5.22	0.001
Fat (%)	3.05 ± 0.87 ^a^	3.83 ± 0.49	3.85 ± 0.60 ^b^	3.96 ± 0.51 ^b^	32.07	<0.001
CLA c9,t11 (%)	0.509 ± 0.192 ^a^	0.664 ± 0.293 ^b^	0.637 ± 0.259 ^b^	0.668 ± 0.291 ^b^	4.86	0.002
CLA t10,c12 (%)	0.0354 ± 0.0451 ^a^	0.0340 ± 0.0410 ^a^	0.0296 ± 0.0282 ^a^	0.0334 ± 0.0417 ^a^	0.69	0.556
C18:1 cis-9 (%)	24.40 ± 4.29 ^a^	23.11 ± 4.53 ^a^	22.98 ± 4.40 ^a^	22.84 ± 3.88 ^a^	1.72	0.162

BCS 1 (low BCS; 2.0–2.5), BCS 2 (moderate-low BCS; 2.75–3.0), BCS 3 (moderate BCS; 3.25–3.5), and BCS 4 (high BCS; 3.75–4.0). BCS, body condition score; CLA, conjugated linoleic acid; CLA c9,t11, cis-9, trans-11 conjugated linoleic acid; CLA t10,c12, trans-10, cis-12 conjugated linoleic acid; C18:1 cis-9, oleic acid (cis-9 octadecenoic acid). Values are presented as mean ± standard deviation (SD). Differences among groups were evaluated using one-way analysis of variance (ANOVA). Statistical significance was declared at *p* < 0.05. Within each row, means not sharing a common superscript letter differ significantly according to Tukey’s HSD post hoc test (*p* < 0.05).

**Table 4:** Effect of adjustment for parity, exact days in milk (DIM), and milk yield on the associations between BCS category and selected discriminatory biomarkers.

Biomarker	Unadjusted F for BCS	Adjusted F for BCS	Change After Adjustment (%)	Adjusted *p*-Value
IL-8	526.59	512.56	−2.6	<0.001
NEFA	192.01	184.09	−4.1	<0.001
GGT	123.80	121.40	−2.7	<0.001
AST	102.03	100.22	−2.7	<0.001

**Table 5:** Construction of composite indices.

Index	Biomarkers Included	Direction of Pathological Change	Biological Interpretation
EDI	NEFA, BHB	↑ NEFA, ↑ BHB	negative energy balance and ketone-body burden
HSI	GGT, AST	↑ GGT, ↑ AST	hepatic metabolic stress
OII	MDA, SOD, TAS	↑ MDA, ↑ SOD, ↓ TAS	oxidative imbalance and reduced antioxidant capacity
IMI	IL-8, SCC, lysozyme, Lp, β-lactoglobulin	↑ all	inflammatory and mammary gland activation
MAI	milk yield, fat, protein, CLA c9,t11	↓ yield, ↓ fat, ↑ protein, ↓ CLA	altered milk production and composition
ILBPS	EDI, HSI, OII, IMI, MAI	↑ all	integrated low-BCS molecular phenotype

AST, aspartate aminotransferase; BHB, β-hydroxybutyrate; CLA, conjugated linoleic acid; EDI, Energy Dysregulation Index; GGT, gamma-glutamyl transferase; HSI, Hepatic Stress Index; IL-8, interleukin-8; ILBPS, Integrated Low-BCS Phenotype Score; IMI, Inflammatory–Mammary Index; Lp, lactoperoxidase; MAI, Milk Alteration Index; MDA, malondialdehyde; NEFA, non-esterified fatty acids; OII, Oxidative Imbalance Index; SCC, somatic cell count; SOD, superoxide dismutase; TAS, total antioxidant status. Arrows indicate the direction associated with adverse physiological change used for index construction.

**Table 6:** Composite molecular phenotype indices according to BCS class.

Index	BCS 1	BCS 2	BCS 3	BCS 4
Energy Dysregulation Index (EDI)	1.72	−0.69	−0.56	−0.47
Hepatic Stress Index (HSI)	1.73	−0.56	−0.56	−0.60
Oxidative Imbalance Index (OII)	1.69	−0.63	−0.59	−0.47
Inflammatory-Mammary Index (IMI)	1.73	−0.55	−0.57	−0.61
Milk Alteration Index (MAI)	1.70	−0.71	−0.30	−0.69
Integrated Low-BCS Phenotype Score (ILBPS)	1.71	−0.63	−0.52	−0.57

BCS 1 (low BCS; 2.0–2.5), BCS 2 (moderate-low BCS; 2.75–3.0), BCS 3 (moderate BCS; 3.25–3.5), and BCS 4 (high BCS; 3.75–4.0).

**Table 7:** PLS-DA model performance for discrimination of BCS classes.

Model	Components	Accuracy	R^2^Y	Q^2^	Interpretation
PLS-DA BCS 1 vs. BCS 2–4	2	0.91	0.82	0.71	strong discrimination
PLS-DA four BCS classes	3	0.76	0.68	0.52	moderate discrimination
Cross-validated model	2	0.88	0.79	0.66	acceptable stability

BCS 1 (low BCS; 2.0–2.5), BCS 2 (moderate-low BCS; 2.75–3.0), BCS 3 (moderate BCS; 3.25–3.5), and BCS 4 (high BCS; 3.75–4.0). BCS, body condition score; PLS-DA, partial least squares discriminant analysis; R^2^Y, explained variance of the response variable; Q^2^, predictive ability estimated by cross-validation. Accuracy represents the proportion of correctly classified observations. Higher R^2^Y and Q^2^ values indicate better model fit and predictive performance, respectively.

**Table 8:** VIP scores from PLS-DA model.

Rank	Biomarker	VIP Score	Biological Pathway	Interpretation
1	IL-8	2.84	inflammation	strongest discriminator
2	NEFA	2.46	energy metabolism	very strong discriminator
3	GGT	2.21	liver function	very strong discriminator
4	SCC	2.05	mammary gland health	very strong discriminator
5	AST	1.94	hepatic/metabolic stress	strong discriminator
6	Lp	1.61	innate immunity	strong discriminator
7	BHB	1.44	energy metabolism	relevant discriminator
8	MDA	1.28	oxidative stress	relevant discriminator
9	TAS	1.12	antioxidant capacity	moderate discriminator
10	Lysozyme	1.05	innate immunity	moderate discriminator
11	Fat (%)	0.96	milk composition	weak/moderate
12	β-Lactoglobulin	0.91	milk protein profile	weak/moderate
13	SOD	0.82	antioxidant response	weak
14	Glucose	0.65	energy metabolism	weak
15	GPx	0.42	antioxidant enzyme	negligible
16	Lactoferrin	0.38	immune protein	negligible
17	CLA t10,c12	0.31	fatty acid profile	negligible
18	C18:1 cis-9	0.27	fatty acid profile	negligible

AST, aspartate aminotransferase; BHB, β-hydroxybutyrate; CLA, conjugated linoleic acid; GGT, gamma-glutamyl transferase; GPx, glutathione peroxidase; IL-8, interleukin-8; Lp, lactoperoxidase; MDA, malondialdehyde; NEFA, non-esterified fatty acids; SCC, somatic cell count; SOD, superoxide dismutase; TAS, total antioxidant status; VIP, Variable Importance in Projection. Variables with VIP > 1.0 were considered important contributors to discrimination among BCS classes, whereas variables with VIP < 0.8 were considered to have limited discriminatory value.

**Table 9:** Discriminatory performance of individual and integrated biomarker models for distinguishing cows with low body condition score (BCS ≤ 2.5) from cows in the remaining BCS categories based on receiver operating characteristic (ROC) analysis.

Model/Biomarker	AUC (95% CI)	Sens.	Spec.	Cut-Off
IL-8	0.989 (0.980–0.996)	100.0%	96.8%	6.39
NEFA	0.973 (0.958–0.985)	96.2%	95.3%	1.25
GGT	0.968 (0.951–0.982)	100.0%	89.2%	125.4
AST	0.964 (0.941–0.982)	96.2%	87.4%	105.9
BHB	0.910 (0.848–0.957)	88.5%	86.3%	1.02
IL-8 + NEFA	0.989 (0.980–0.995)	100.0%	97.3%	0.162
IL-8 + NEFA + GGT	0.990 (0.982–0.996)	100.0%	97.3%	0.228
IL-8 + NEFA + AST	0.989 (0.980–0.995)	100.0%	97.1%	0.146
IL-8 + NEFA + GGT + AST	0.990 (0.982–0.996)	100.0%	97.3%	0.225

**Table 10:** Sensitivity analysis comparing the discriminatory performance of equal-weight and VIP-weighted Integrated Low-BCS Phenotype Scores (ILBPS) for distinguishing cows with low body condition score (BCS 1) from cows in BCS categories 2–4.

Score	BCS 1 Mean ± SD	BCS 2 Mean ± SD	BCS 3 Mean ± SD	BCS 4 Mean ± SD	AUC	Sensitivity (%)	Specificity (%)	Optimal Cut-Off
Equal-weight ILBPS	1.319 ± 0.367	−0.138 ± 0.211	−0.102 ± 0.231	−0.162 ± 0.204	0.980	97.0	97.8	0.662
VIP-weighted ILBPS	1.390 ± 0.394	−0.140 ± 0.212	−0.111 ± 0.222	−0.174 ± 0.198	0.980	98.0	97.6	0.471

BCS 1, low body condition score (2.0–2.5); BCS 2, moderate-low BCS (2.75–3.0); BCS 3, moderate BCS (3.25–3.5); BCS 4, high BCS (3.75–4.0); AUC, area under the receiver operating characteristic curve; ILBPS, Integrated Low-BCS Phenotype Score; VIP, Variable Importance in Projection. The difference between the AUCs of the equal-weight and VIP-weighted ILBPS was evaluated using DeLong’s test for correlated ROC curves and was not statistically significant (*p* = 0.854).

**Table 11:** Pearson correlation coefficients among metabolic, oxidative stress, inflammatory, mammary gland, and milk production biomarkers.

Variable	Glucose	NEFA	BHB	AST	GGT	MDA	TAS	SOD	GPx	IL-8	SCC	Lysozyme	Lactoferrin	Lactoperoxidase	β-Lactoglobulin	Milk Yield	Fat	Protein
**Glucose**	1.000																	
**NEFA**	−0.41 ***	1.000																
**BHB**	−0.37 ***	0.76 ***	1.000															
**AST**	−0.28 ***	0.64 ***	0.58 ***	1.000														
**GGT**	−0.24 **	0.58 ***	0.51 ***	0.73 ***	1.000													
**MDA**	−0.30 ***	0.63 ***	0.57 ***	0.66 ***	0.62 ***	1.000												
**TAS**	0.34 ***	−0.59 ***	−0.50 ***	−0.52 ***	−0.46 ***	−0.72 ***	1.000											
**SOD**	0.29 ***	−0.49 ***	−0.42 ***	−0.43 ***	−0.38 ***	−0.65 ***	0.71 ***	1.000										
**GPx**	0.25 **	−0.45 ***	−0.37 ***	−0.36 ***	−0.32 ***	−0.56 ***	0.64 ***	0.69 ***	1.000									
**IL−8**	−0.32 ***	0.70 ***	0.63 ***	0.64 ***	0.60 ***	0.65 ***	−0.56 ***	−0.46 ***	−0.40 ***	1.000								
**SCC**	−0.27 ***	0.66 ***	0.58 ***	0.59 ***	0.56 ***	0.60 ***	−0.51 ***	−0.42 ***	−0.36 ***	0.82 ***	1.000							
**Lysozyme**	−0.24 **	0.58 ***	0.47 ***	0.49 ***	0.45 ***	0.50 ***	−0.45 ***	−0.38 ***	−0.32 ***	0.68 ***	0.72 ***	1.000						
**Lactoferrin**	−0.26 ***	0.59 ***	0.50 ***	0.52 ***	0.46 ***	0.51 ***	−0.48 ***	−0.40 ***	−0.34 ***	0.71 ***	0.66 ***	0.64 ***	1.000					
**Lactoperoxidase**	−0.25 **	0.60 ***	0.51 ***	0.54 ***	0.49 ***	0.53 ***	−0.50 ***	−0.41 ***	−0.35 ***	0.72 ***	0.69 ***	0.66 ***	0.68 ***	1.000				
**β-Lactoglobulin**	−0.21 **	0.51 ***	0.44 ***	0.46 ***	0.42 ***	0.43 ***	−0.40 ***	−0.33 ***	−0.29 ***	0.60 ***	0.57 ***	0.54 ***	0.58 ***	0.63 ***	1.000			
**Milk yield**	0.43 ***	−0.63 ***	−0.51 ***	−0.55 ***	−0.48 ***	−0.58 ***	0.56 ***	0.45 ***	0.39 ***	−0.65 ***	−0.60 ***	−0.54 ***	−0.57 ***	−0.58 ***	−0.51 ***	1.000		
**Fat**	0.36 ***	−0.57 ***	−0.48 ***	−0.49 ***	−0.43 ***	−0.53 ***	0.48 ***	0.38 ***	0.33 ***	−0.58 ***	−0.53 ***	−0.46 ***	−0.48 ***	−0.51 ***	−0.45 ***	0.76 ***	1.000	
**Protein**	0.34 ***	−0.48 ***	−0.39 ***	−0.41 ***	−0.37 ***	−0.42 ***	0.37 ***	0.29 ***	0.24 **	−0.47 ***	−0.44 ***	−0.38 ***	−0.41 ***	−0.43 ***	−0.37 ***	0.69 ***	0.62 ***	1.000

Significance levels: ** *p* < 0.01; *** *p* < 0.001.

**Table 12:** Comparison of pooled Pearson correlations and partial correlations adjusted for body condition score for selected biomarker relationships.

Biomarker Pair	Pooled Pearson r	BCS-Adjusted Partial r
IL-8—SCC	0.82	0.58
AST—GGT	0.73	0.61
NEFA—BHB	0.68	0.52
NEFA—GGT	0.62	0.41
NEFA—AST	0.58	0.38
NEFA—IL-8	0.61	0.34

BCS, body condition score; AST, aspartate aminotransferase; BHB, β-hydroxybutyrate; GGT, gamma-glutamyl transferase; IL-8, interleukin-8; NEFA, non-esterified fatty acids; SCC, somatic cell count. Partial correlation coefficients were calculated after adjustment for BCS category.

**Table 13:** Group-specific characteristics of cows according to body condition score category.

Variable	BCS 1 (n = 51)	BCS 2 (n = 178)	BCS 3 (n = 239)	BCS 4 (n = 112)	*p*-Value
Parity, n (%)					0.149
2nd lactation	19 (37.3)	88 (49.4)	125 (52.3)	63 (56.3)	
3rd lactation	32 (62.7)	90 (50.6)	114 (47.7)	49 (43.8)	
Exact DIM, days	55.9 ± 5.7	55.1 ± 5.8	54.4 ± 5.6	54.7 ± 5.7	0.072
Body weight, kg	605 ± 51	628 ± 53	637 ± 52	644 ± 54	0.008
Milk yield, kg/d	28.54 ± 7.74	34.01 ± 7.14	32.42 ± 8.41	33.19 ± 8.95	<0.001

Values are presented as mean ± SD or n (%). Parity distribution was compared among BCS categories using the chi-square test, whereas continuous variables were evaluated using one-way ANOVA. BCS, body condition score; DIM, days in milk.

**Table 14:** Ingredient composition, feeding characteristics, and nutritional and production-related parameters of the total mixed ration (TMR) offered to dairy cows during the study period (March–October).

Item	Value	Unit
Ingredient composition		
Maize silage	11.05	kg DM/d
Alfalfa silage	3.50	kg DM/d
Ensiled maize grain	2.50	kg DM/d
Soybean meal	2.50	kg DM/d
Rapeseed meal	2.10	kg DM/d
Pasture ground chalk	0.20	kg DM/d
Salt	0.05	kg DM/d
Magnesium oxide	0.06	kg DM/d
Feeding and nutritional characteristics		
Total dry matter	21.20	kg/d
Daily dry matter intake	19.90	kg/d
Net energy for lactation (NE_l_)	1.75	Mcal/kg
Feeding frequency	2	times/d
Production-related characteristics		
Average milk production	37.02	kg/d
Unit of milk production balance	3.45	%
Protein digested in the small intestine when rumen-fermentable nitrogen is limiting	2.51	*a*
Protein digested in the small intestine when rumen-fermentable energy is limiting	2.23	*a*

DM, dry matter; TMR, total mixed ration; NE_l_, net energy for lactation. Diets were formulated according to the INRA feeding system and offered ad libitum. The TMR formulation remained unchanged throughout the study period from March to October. *a* The unit was not specified in the available ration formulation record.

## Data Availability

All data generated or analyzed during the study are included within the article.

## Abbreviations

The following abbreviations are used in this manuscript:

**Abbreviation**	**Definition**
ANOVA	Analysis of Variance
AP-1	Activator Protein-1
AST	Aspartate Aminotransferase
BCS	Body Condition Score
BHB	β-Hydroxybutyrate
BLG	β-Lactoglobulin
CLA	Conjugated Linoleic Acid
DIM	Days in Milk
EDI	Energy Dysregulation Index
ELISA	Enzyme-Linked Immunosorbent Assay
FAME	Fatty Acid Methyl Esters
GGT	Gamma-Glutamyl Transferase
GPx	Glutathione Peroxidase
GSH	Reduced Glutathione
GSSG	Oxidized Glutathione
HSI	Hepatic Stress Index
IL-8	Interleukin-8
ILBPS	Integrated Low-BCS Phenotype Score
IMI	Inflammatory–Mammary Index
LF	Lactoferrin
Lp	Lactoperoxidase
LZ	Lysozyme
MAI	Milk Alteration Index
MDA	Malondialdehyde
NEB	Negative Energy Balance
NEFA	Non-Esterified Fatty Acids
NF-κB	Nuclear Factor Kappa B
NLRP3	NLR Family Pyrin Domain Containing 3
OII	Oxidative Imbalance Index
PCA	Principal Component Analysis
PLS-DA	Partial Least Squares Discriminant Analysis
Q^2^	Predictive Ability
ROS	Reactive Oxygen Species
RP-HPLC	Reversed-Phase High-Performance Liquid Chromatography
R^2^Y	Explained Variance of the Response Variable
SCC	Somatic Cell Count
SD	Standard Deviation
SOD	Superoxide Dismutase
TAS	Total Antioxidant Status
TBARS	Thiobarbituric Acid Reactive Substances
TLR	Toll-Like Receptor
TMR	Total Mixed Ration
VIP	Variable Importance in Projection
WULS-SGGW	Warsaw University of Life Sciences
